# Single molecule fate of HIV-1 envelope reveals late-stage viral lattice incorporation

**DOI:** 10.1038/s41467-018-04220-w

**Published:** 2018-05-10

**Authors:** Carmen A. Buttler, Nairi Pezeshkian, Melissa V. Fernandez, Jesse Aaron, Sofya Norman, Eric O. Freed, Schuyler B. van Engelenburg

**Affiliations:** 1 Molecular and Cellular Biophysics Program, Department of Biological Sciences, University of Denver, Denver, CO 80210 USA; 2 HIV Dynamics and Replication Program, Center for Cancer Research, National Cancer Institute, Frederick, MD 21702 USA; 3 Advanced Imaging Center, Janelia Research Campus, Howard Hughes Medical Institute, Ashburn, VA 20147 USA

## Abstract

*Human immunodeficiency virus type 1* (HIV-1) assembly occurs on the inner leaflet of the host cell plasma membrane, incorporating the essential viral envelope glycoprotein (Env) within a budding lattice of HIV-1 Gag structural proteins. The mechanism by which Env incorporates into viral particles remains poorly understood. To determine the mechanism of recruitment of Env to assembly sites, we interrogate the subviral angular distribution of Env on cell-associated virus using multicolor, three-dimensional (3D) superresolution microscopy. We demonstrate that, in a manner dependent on cell type and on the long cytoplasmic tail of Env, the distribution of Env is biased toward the necks of cell-associated particles. We postulate that this neck-biased distribution is regulated by vesicular retention and steric complementarity of Env during independent Gag lattice formation.

## Abstract (web summary)

HIV particles contain a relatively low amount of viral envelope (Env), but underlying packaging mechanisms are poorly understood. Here, the authors use superresolution microscopy and show that Env distribution is biased toward the necks of cell-associated particles during assembly.

## Introduction

Virus assembly involves a choreographed coalescence of viral and host biomolecules to create new infectious particles, which propagate infection. In the case of HIV-1 assembly, the structural polypeptide Gag anchors to the inner leaflet of the plasma membrane through the matrix (MA) domain and oligomerizes to create a lattice which deforms the membrane. The HIV-1 Env glycoprotein complex traffics through the secretory pathway to the plasma membrane, where it is displayed as a heterotrimer composed of three molecules each of the surface glycoprotein gp120 and the transmembrane glycoprotein gp41^1–3^. Determinants driving Gag and Env to efficiently co-assemble remain unclear, but numerous studies have implicated the long cytoplasmic tail of gp41 (Env-CT) in virus particle incorporation^4–8^.

Further compounding the complexity of HIV-1 assembly is the relative sparsity of Env on individual released particles (7–14 trimers)^9^. This suggests that Env incorporation into nascent Gag lattices is tightly regulated, but the mechanisms of regulation are also poorly understood. Specific Env retention at the virus assembly site is believed to be due to steric trapping of the long Env-CT between hexamers of Gag-MA trimers^10–13^. In support of this model, a small deletion in the second predicted helix of Env-CT (LLP-3), *d8*, imposes Env incorporation defects that can be rescued by complementary mutations in the Gag-MA domain^14,15^. Furthermore, deletion of the Env-CT (*CTΔ144*) results in a reduction in virus incorporation of Env, but this reduction is cell-type-dependent^5,6,16^, suggesting that host cell factors regulate HIV-1 assembly.

Herein, we demonstrate that steric trapping fixes the angular distribution of Env clusters at virus budding sites, thereby driving the incorporation of Env into assembling HIV-1 particles. We show that Env encounters the Gag lattice late in lattice assembly and that this is cell-type-dependent as well as dependent on the Env-CT.

## Results

### Measurement of Env angular distribution at assembly sites

**Fig. 1:**
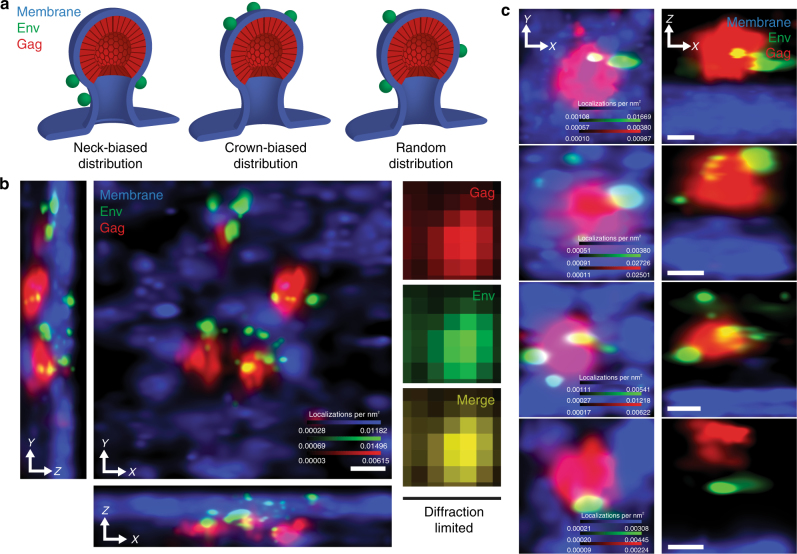
Multicolor three-dimensional superresolution microscopy enables quantitative interrogation of angular distributions of Env at HIV-1 assembly sites. **a** Hypothetical models for angular spatial arrangements of Env clusters around a viral lattice (plasma membrane, blue; Env, green; Gag, red). Neck-distributed Env clusters would result from late Env acquisition, following Gag lattice assembly (left). Crown-distributed Env clusters would result from assembly of Gag at sites of Env clustering (middle). Unbiased distributions of Env clusters are expected if Env is stochastically trapped throughout the lifetime of Gag lattice assembly (right). **b** Superresolution imaging in 3D resolves individual Env clusters (green) decorating four virus assembly sites (Gag, red) protruding from the plasma membrane (blue; center *X*–*Y* projection and lower and left *Z* (optical axis) projections). Diffraction-limited microscopy fails to resolve these budding events (right images, *X*–*Y* projection). Scale bar is 100 nm. Diffraction-limited pixel size is 133 nm. **c** Representative HIV-1 assembly sites segmented from CEM-A cells, observed by projection (*X*–*Y*, left) and perpendicular (*X*–*Z*, right) views. Scale bars are 100 nm

We hypothesized that, by interrogating the angular distribution of Env on the surfaces of cell-associated virus particles, we could determine when Env encounters the Gag lattice (Fig. 1a). To determine if this timing is dictated by host cell factors, we employed two cell lines: CEM-A, a T-cell line permissive for HIV-1 replication^17^, and COS7, a fibroblast-like cell line. In both cell types, single-round infection with VSV-G pseudotyped virus (NL4–3 reference genome) allowed for expression of native levels of Gag and Env. Infection was performed with particles deficient for protease (*ΔPol*) in order to prevent premature processing of the cell-associated Gag lattice and to keep the angular distribution of Env about the budding particle surface fixed. Release-deficient virus containing a late-domain mutation (*ΔPTAP*)^18–20^ was used to arrest particles at a late stage of budding, strictly limiting analysis to cell-associated particles. Collectively, this system enables production of HIV-1 assembly sites for which the budding axis can be identified and used to measure the angular arrangement of Env.

Three-dimensional superresolution imaging of infected cells was achieved using interferometric photo-activated localization microscopy (iPALM)^21,22^, which allowed us to localize both Gag and Env molecules on a subviral scale (with a localization precision of 10–20 nm; Supplementary Fig. 1), along with the host cell plasma membrane, which was mapped by co-expression of a myristoylated photoswitchable fluorescent protein^23^ (S15-PSCFP2; Fig. 1b). Single assembly sites were segmented for analysis if a cluster of Gag was proximal to Env clusters, the local plasma membrane was sufficiently sampled, and the segmented area was resolved from other assembly sites (see “Methods” section, Supplementary Fig. 2). Gag cluster centroids were estimated as described previously^22^ (Supplementary Fig. 3). Env clusters that were localized less than a particle radius from the Gag centroid and residing extracellularly relative to the local plasma membrane were classified as assembly site proximal. Principle component analysis of the local plasma membrane topography at each assembly site (~200 × 200–400 × 400 nm) was used to define the viral budding axis, to which the angular positions of Env clusters could be referenced, (Fig. 1c and Supplementary Figs. 4 and 5) and to confirm that bud neck lengths were consistent with cell-associated particles (Supplementary Fig. 6). Due to the sparsity of Env on individual particles^9^, we derived statistical power from single-particle averaging of hundreds of aligned individual HIV-1 assembly sites. Single assembly site averaging was used to determine the probability density of Env with respect to the elevation angle (*φ*), defined relative to the virus bud equator. We tested this approach using simulated data mimicking the three models presented in Fig. 1a and showed that bias of Env in the neck or crown of the particles is readily detectable (Supplementary Figs. 7). We additionally employed an orthogonal approach to explicitly measure the angular position (*φ*) of individual Env clusters at single virus assembly sites (Supplementary Figs. 8 and 9).

In order to assess the uncertainty in measurement of the angular probability distribution for Env, we used Monte Carlo simulation to generate synthetic virus assembly sites using empirically derived seed values (see Methods section). By analysis of this simulated data set, we estimate our angular uncertainty to be ~4.39 ± 0.12° (s.d. ± s.e.m., *n* = 2500; Supplementary Fig. 10). To complement the error estimates from simulation data, we devised an additional analysis method utilizing phase correlation between the membrane channels of aligned random half data sets to estimate that our plane fitting method resulted in no more than 3° of uncertainty in rotational alignment (Supplementary Fig. 11), which is comparable to our simulated error in Env angular measurements. Phase correlation between the Env channels of random half data sets suggested, on average, 9° of uncertainty in angular sampling of Env (*σ*_*φ*_) on the surfaces of virus particles (Supplementary Fig. 12). These results collectively suggest that a conservative upper limit for the angular resolution of our system is 9°.

**Fig. 2:**
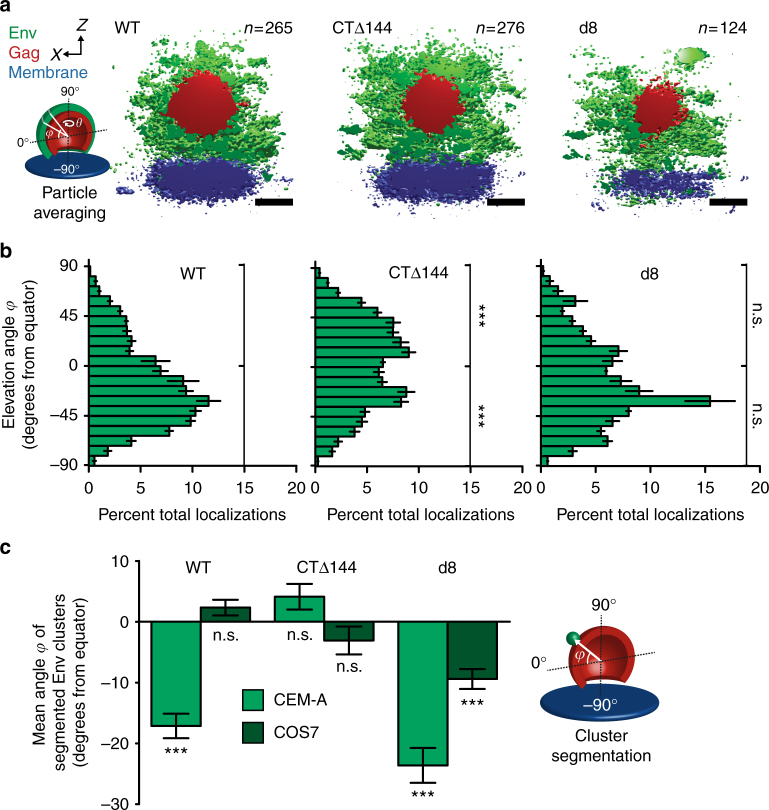
The angular distribution of Env on the surfaces of virus particles is dependent on cell type and the Env-CT. **a** Cross-sections of aligned and averaged virus assembly sites produced in CEM-A cells (WT, left, *n* = 265 particles from three cells; *CTΔ144*, center, *n* = 276 particles from four cells; *d8*, right, *n* = 124 particles from four cells). Isosurface thresholds are 1.0 × 10^−3^ (Env, green), 3.0 × 10^−3^ (Gag, red), and 4.5 × 10^−3^ (plasma membrane, blue) localizations × nm^−3^. Scale bars are 100 nm. **b** Probability distributions of WT- (left), CTΔ144- (center), and d8-Env (right) about averaged clusters produced in CEM-A. The elevation angle (*φ*) was binned in 9° increments, according to the estimated sampling resolution (Supplementary Fig. 12). Error bars indicate s.d. of three pairs of random half data sets. Upper and lower hemispheres were integrated, error from each bin was added in quadrature, and probabilities were *t*-tested against WT-Env. **c** The mean angular distributions of each Env genotype were measured explicitly from clusters of Env localizations on individual virus assembly sites for both cell types tested (see distributions in Supplementary Fig. 14). Error bars indicate s.e.m. Mean angular probabilities were tested for significant differences from a normal distribution centered at 0°. ***P < 0.0001 and n.s. not significant by two-way ANOVA and Bonferroni post-test

Probability densities of averaged wild-type (WT) Env showed that the T-cell line, CEM-A, produces virus assembly sites with an angular distribution biased toward the necks of budding particles (Fig. 2a, b and Supplementary Fig. 13). Herein, we defined the elevation angle (*φ*) at the equator of the particle as 0° (orthogonal to the budding axis), the neck of the particle (southern hemisphere) from 0° to −90°, and the crown of the particle (northern hemisphere) from 0° to 90° (Fig. 2a cartoon). Using our orthogonal approach of directly measuring individual Env clusters at single assembly sites, we observed comparable angular bias of Env toward virus necks, with a mean value of *φ* = −17.1 ± 2.0° (s.e.m., *n* = 338) and a skewness of 0.523 relative to the equator of the particle (Fig. 2c and Supplementary Fig. 14). In contrast, particles produced in the COS7 fibroblast-like cell line showed an unbiased angular distribution of Env using the single-particle averaging approach (Supplementary Fig. 13), in agreement with a mean *φ* = 2.3 ± 1.3° (*n* = 813) and a skewness of −0.0970 obtained by individual cluster measurements (Fig. 2c and Supplementary Table 1). Attempts to confirm these observations by immunogold transmission electron microscopy failed to produce robust statistical sampling due to low-density Env labeling and frequent off-axis sectioning of budding profiles (Supplementary Fig. 15).

Next, we tested whether the Env-CT was responsible for the neck bias of Env in the CEM-A cell line. Indeed, removal of the Env-CT (*CTΔ144*) produced an unbiased distribution of Env signal on the surfaces of budding particles (mean *φ* = 4.1 ± 2.1°, *n* = 367; skewness −0.221). These results show that the Env-CT is responsible for the neck bias measured for WT-Env (Fig. 2 and Supplementary Figs. 13 and 14). As expected, the *CTΔ144* mutation had no effect on the already unbiased distribution of Env in particles produced by COS7 (mean *φ* = -3.07 ± 2.3°, *n* = 260; skewness −0.0279).

We next sought to determine if inhibition of Gag lattice trapping could induce a neck bias for particles produced in the COS7 cell line. Strikingly, deletion of residues in the Env-CT (*d8*) that lead to steric clashing with the Gag-MA lattice and reduced Env incorporation^14^ resulted in a neck-biased distribution of Env in COS7 cells (mean *φ* = −9.4 ± 1.6°, *n* = 582; skewness 0.236), where no bias was observed for WT-Env. These results show that steric clashing imposed by the *d8* mutation relegates Env to the periphery of the budding Gag lattice in COS7 cells (Fig. 2 and Supplementary Figs. 13 and 14). As anticipated, the *d8* mutation did not produce a significant change in the Env neck-distributed phenotype observed for WT-Env in CEM-A cells (mean *φ* = −23.6 ± 2.9°, *n* = 134; skewness 0.530).

### Single-molecule tracking of Env diffusivity on cell surfaces

**Fig. 3:**
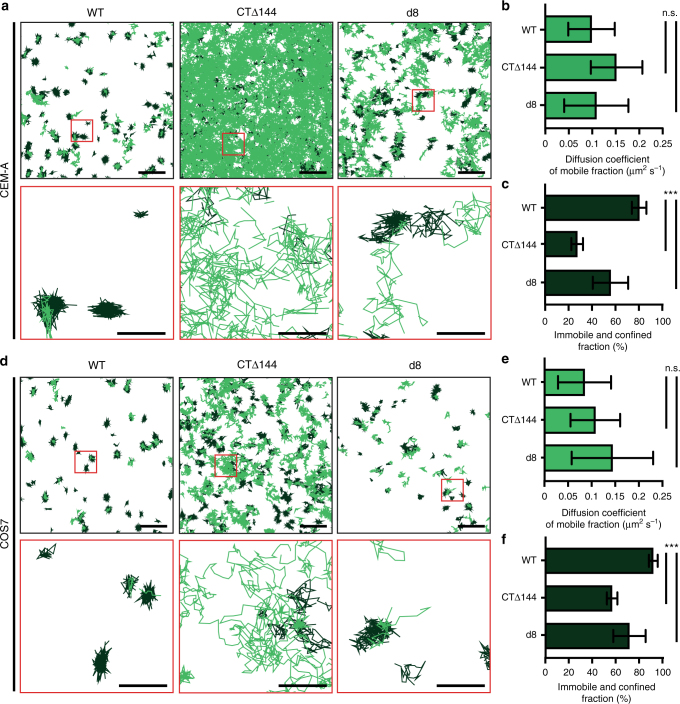
Single-molecule tracking of HIV-1 Env trimers reveals that the Env-CT does not significantly alter diffusivity on the nanoscale, but determines the fraction of Env that is confined/immobile. **a**,** d** Single-particle tracking of WT-Env (left), CTΔ144-Env (middle), and d8-Env (right) labeled with Fab b12-Atto565 in **a** CEM-A cells and **d** COS7 cells. Scale bars are 2 μm (top) and 500 nm (red inset region, bottom). Tracks were classified as immobile and confined (dark green) versus mobile (light green). **b**,** e** Mean diffusion coefficients were computed based on individual molecular tracks in CEM-A (WT-Env, *n* = 1514; CTΔ144-Env, *n* = 9694; and d8-Env, *n* = 2139, each from four biological replicates) and COS7 (WT-Env, *n* = 274; CTΔ144-Env, *n* = 2238; and d8-Env, *n* = 1019 each from ≥4 biological replicates). **b** In CEM-A cells, the mean diffusion coefficients of tracks classified as mobile for the CTΔ144-Env (*D*_mobile_ = 0.149 ± 0.054 μm^2^ s^−1^) and d8-Env (*D*_mobile_ = 0.107 ± 0.067 μm^2^ s^−1^) are not significantly different when compared to WT-Env (*D*_mobile_ = 0.097 ± 0.049 μm^2^ s^−1^). **e** For COS7 cells, mean diffusion coefficients of tracks classified as mobile indicate that the *CTΔ144* (*D*_mobile_ = 0.108 ± 0.053 μm^2^ s^−1^) and *d8* mutations (*D*_mobile_ = 0.144 ± 0.087 μm^2^ s^−1^) do not significantly alter the diffusion rates of Env when compared to WT-Env (*D*_mobile_ = 0.085 ± 0.056 μm^2^ s^−1^). **c**,** f** The Env-CT dictates the confined/immobile fraction of Env on the plasma membrane. **c** In CEM-A cells, the confined/immobile fractions of the CTΔ144-Env and (27 ± 5%) and d8-Env (56 ± 15%) were both found to be significantly smaller relative to WT-Env (80 ± 6%). **f** In COS7 cells, the confined/immobile fractions of the CTΔ144-Env (57 ± 5%) and d8-Env (72 ± 13%) were significantly lower relative to WT-Env (92 ± 4%), suggesting that steric trapping of Env is dependent on the Env-CT. Error bars indicate s.d. ***P < 0.0001 and n.s. indicates not significant using one-way ANOVA with Tukey’s post-test. CEM-A (WT-Env, *n* = 7862; CTΔ144-Env, *n* = 13,338; and d8-Env, *n* = 5434) and COS7 (WT-Env, *n* = 3790; CTΔ144-Env, *n* = 5283; and d8-Env, *n* = 4384)

Given that removal of the Env-CT (CTΔ144-Env) led to an unbiased angular distribution, we hypothesized that CTΔ144-Env may not be trapped by the Gag lattice, leading to sampling of the entire bud surface by diffusing Env trimers. This lack of trapping would also account for the substantial incorporation defect we observed with this genotype in both cell types (Supplementary Fig. 16). To test this hypothesis, we performed single-molecule tracking of WT-, CTΔ144-, and d8-Env trimers on the surfaces of CEM-A and COS7 cells using an anti-Env Fab fragment (b12), conjugated to Atto565 dye, in order to interrogate the nanoscale dynamics of these molecules during virus assembly (Fig. 3). We imaged Env on sparsely labeled cells, measured single-molecule positions, and used the uTrack software^24^ to link single-molecule trajectories and compute the diffusion coefficients of each track (Supplementary Fig. 17). Individual tracks were then classified as either mobile or confined/immobile, based on the estimated slope of the moment scaling spectrums In CEM-A cells, we found that the mean diffusion coefficients of WT-, CTΔ144-, and d8-Env tracks classified as mobile did not significantly differ from each other, with mean diffusion coefficients of 0.097 ± 0.049 (s.d., *n* = 1514 across four biological replicates), 0.149 ± 0.054 (*n* = 9694 across four biological replicates), and 0.107 ± 0.067 μm^2^ s^−1^ (*n* = 2139 across four biological replicates), respectively (Fig. 3b), agreeing well with published results of cell-associated WT- and CTΔ144-Env diffusion using an orthogonal technique^11^. However, the proportion of tracks classified as confined/immobile was significantly higher for WT-Env (weighted mean of 80 ± 6% confined/immobile tracks across four biological replicates, s.d., *n* = 7862 tracks) when compared with both CTΔ144- (27 ± 5% confined/immobile, *n* = 13,338 tracks) and d8-Env (56 ± 15% confined/immobile, *n* = 5434 tracks, Fig. 3c), in agreement with previous microscale FRAP data for WT- and CTΔ144-Env^12^. Our single-molecule measurements of Env dynamics suggest that the reduced incorporation of CTΔ144- and d8-Env into virus assembly sites derives from a reduced likelihood of steric trapping within Gag lattices, rather than from differential diffusion behavior. In COS7 cells, mean diffusion coefficients of Env did not significantly differ from those of Env in CEM-A cells, and the same trend of mobile versus immobilized populations was observed between genotypes (Fig. 3d–f), suggesting that the observed differences in diffusion behavior are not cell-type-dependent and do not, on their own, account for the cell-type-dependent differences in Env angular distributions measured by superresolution.

### Intracellular retention correlates to Env distribution bias

**Fig. 4:**
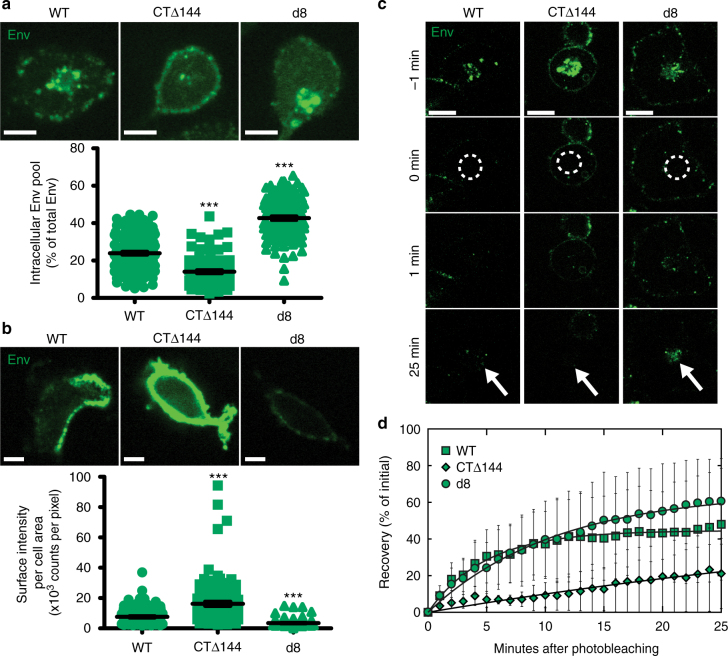
Intracellular retention of Env in CEM-A cells correlates with angular distributions of Env at assembly sites. **a** Pulse-chase-labeled Env (anti-Env Fab b12-Atto565; 15 min) demonstrates greater intracellular accumulation of d8-Env (43 ± 1%, *n* = 123 cells) and WT-Env (24 ± 1%, *n* = 142 cells) relative to CTΔ144-Env (14 ± 1%, *n* = 99 cells) in CEM-A cells. Scale bars are 10 μm (representative images above). **b** Labeling of fixed cells with anti-Env 2G12- and b12-AF647 probes demonstrates reduced levels of surface-exposed WT- and d8-Env relative to CTΔ144-Env. Scale bars are 10 µm (representative images above). **a**, **b** Bar and error bars represent mean and s.e.m. **c** Representative FRAP time-lapse highlighting the rates of recovery of intracellular Env pools. Time points are relative to photobleaching (bleach region, white dashed circle). Scale bars are 20 μm. **d** FRAP experiments demonstrate reduced internal compartment recovery rates for CTΔ144-Env (0.016 ± 0.01 s^−1^) relative to WT-Env (0.21 ± 0.01 s^−1^, relative to CTΔ144-Env *P* < 0.0001) and d8-Env (0.092 ± 0.01 s^−1^, relative to CTΔ144-Env *P* = 0.0036). Error bars indicate s.d. (*n* = 4 cells per genotype). *** indicates significance from WT with *P* < 0.0001. All statistical tests used a two-tailed unpaired *t*-test

**Fig. 5:**
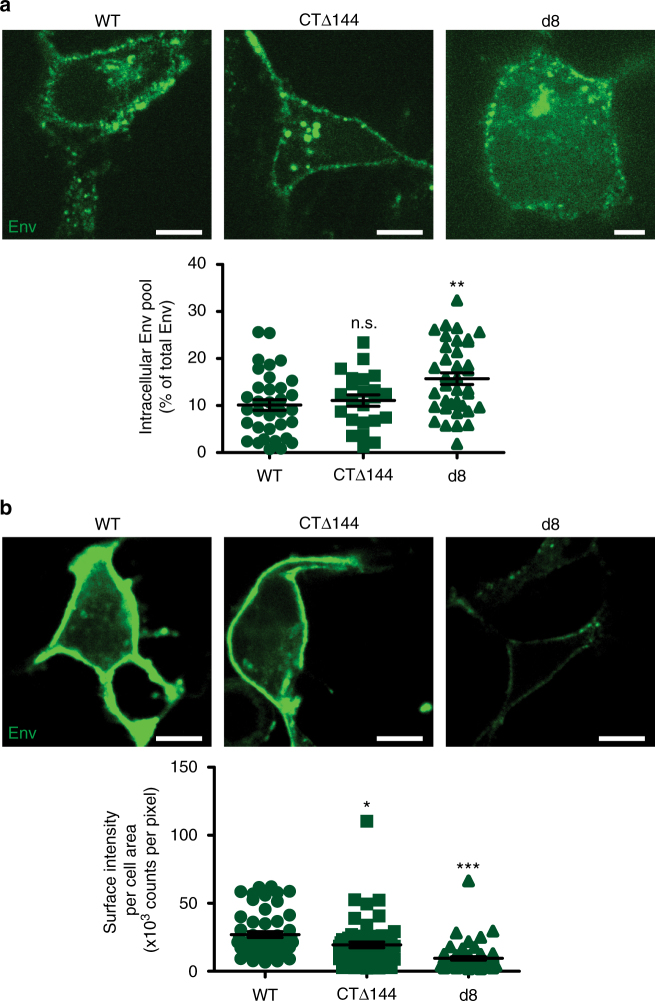
Surface labeling of Env in COS7 cells shows cell-type dependence of Env-CT trafficking and intracellular levels. Infected cells were either fixed directly and surface labeled for Env or pulse-chase labeled live to highlight endocytosed Env pools using the full b12 and 2G12 IgG's conjugated to AF647 or monovalent Fab b12-Atto565, respectively. **a** Representative examples of pulse-chase labeled COS7 cells expressing WT- (left), CTΔ144- (middle), and d8-Env (right) (above). Scale bars are 20 μm. Comparison of the intracellular levels of Env normalized to the total Env signal of each cell (below). There was no significant difference between WT- (*n* = 35) and CTΔ144-Env (*n* = 23), while the intracellular fraction of d8-Env (*n* = 36) was significantly greater than in WT. Bar and error bars indicate mean and s.e.m. **b** Representative equatorial slices (above) from COS7 cells expressing WT- (left), CTΔ144- (middle), and d8-Env (right), labeled for surface-exposed Env only (above panel). Scale bars are 20 μm. Quantified comparison of surface staining intensities for WT- (*n* = 54), CTΔ144- (*n* = 61), and d8-Env (*n* = 57) normalized to cell area (bottom). These results show that in COS7 cells, significantly less Env is displayed on the cell surface for both mutants compared to WT. Bar and error bars represent mean and s.e.m. ***P < 0.0001, **P = 0.0058, and *P *=* 0.0156 represent significance or n.s. not significant from WT-Env as assessed by two-tailed unpaired *t*-test

We hypothesized that the cell-type-dependent neck-biased phenotype observed with WT-Env in CEM-A cells may result from Env retention within intracellular compartments while Gag autonomously assembles on the plasma membrane. To test this hypothesis, we performed live cell pulse-chase labeling of surface-exposed Env with an anti-Env Fab (b12) conjugate to determine the relative levels of internalization of Env and associated mutants. In addition, we also performed fixed cell surface staining of Env to compare relative surface-exposed populations for each genotype. Spinning disk confocal microscopy revealed a large steady-state population of endocytosed Env, which localized with recycling endosomes co-labeled with fluorescent transferrin (Supplementary Fig. 18). We observed a reduction in the intracellular pool and higher levels of CTΔ144-Env on the plasma membrane in CEM-A relative to WT-Env (Fig. 4a, b), consistent with a role for the Env-CT in Env endocytosis^25^. In COS7 cells, however, the intracellular pool did not differ significantly between WT-Env and CTΔ144-Env, and surface levels of WT-Env were slightly higher than CTΔ144-Env, suggesting that endocytosis or intracellular retention of Env is less prevalent in this cell line (Fig. 5). In contrast, the *d8* mutation led to an increase in the intracellular pool of Env relative to WT and lower levels on the plasma membrane in both CEM-A and COS7 cells (Figs. 4a, b and 5).

Given that the steady-state levels of Env mutants show drastic differences, we next quantified the rates of Env internalization and recycling to the plasma membrane by performing fluorescence recovery after photobleaching (FRAP) on the intracellular pool of Env in CEM-A cells. We found that internal pools of CTΔ144-Env recovered at rates more than tenfold slower than WT-Env and more than fivefold slower than d8-Env (Fig. 4c, d), confirming that impairment of endocytosis and lattice incorporation alter the flux of Env intracellular trafficking. The total percent recovery of CTΔ144-Env was considerably lower than either WT- or d8-Env; however, the intracellular pool of d8-Env recovered to a similar extent as WT-Env, despite having a smaller pool of un-bleached plasma membrane Env that could be endocytosed (Fig. 4b–d). This result suggests that larger intracellular pools of d8-Env may result not only from endocytosis of a larger population of freely diffusing Env trimers, but also from an accumulation of endocytosed trimers that are unable to properly return to the plasma membrane (Figs. 4a and 5a).

## Discussion

Collectively, our experimental results demonstrate a complex spatiotemporal pathway for HIV-1 Env incorporation into assembling Gag lattices. Our single-molecule approach, specifically imaging the subviral angular distribution of native levels of Env on the surface of nascent budding HIV-1 particles, enabled accurate quantification (at ~9° resolution) of a neck-biased phenotype present on particles produced in the T-cell line CEM-A, but not on those produced in the fibroblast-like line COS7. In this study, we chose to use a late-domain mutant Gag to trap and preserve the angular orientation of Env during virus budding, as particle release would result in scrambling of the angular distribution of Env. A potential consequence of using release-deficient Gag is that late-domain mutants will create assemblies which continue to polymerize and overfill the particle. Additional Gag lattice polymerization will act to fix Env trimers that partition into the neck of the virus bud. If stochastic trapping of Env trimers was enhanced with late-domain mutant Gag lattices, we would expect to observe a larger number of Env clusters by superresolution imaging than previous studies observing released virus (only 1–4 clusters per particle)^26^. Finally, we were able to measure statistically significant differences in the angular distributions of Env mutants relative to WT-Env as well as observe cell-type differences, suggesting that mutant late-domain assembly sites do not dominantly determine Env distributions.

The measured neck bias of WT-Env in CEM-A T cells suggests that Gag lattices on average begin to form prior to Env encounter, statistically relegating trapped Env to the periphery of assembling lattices and limiting the amount of Env that can be incorporated prior to particle abscission. Strikingly, with assembling particles produced in COS7 cells, the timing of Env acquisition appears to be more synchronous with Gag lattice formation, resulting in an unbiased angular distribution about the viral particle surface, and this discrepancy suggests a role for host cell factors in regulating the timing of encounters. We show that the temporal regulation of Env incorporation is dependent on the presence of the long Env-CT, without which CTΔ144-Env trimers appear to freely diffuse through Gag lattices, enabling continued access to high-angular spaces in the head of the budding particle.

Our results further demonstrate that the angular distribution of Env can be engineered by the introduction of mutations in the Env-CT (*d8* mutation^14^) that create a steric clash between Env and Gag, thereby consigning Env to the periphery of the Gag lattice independent of cell type. These results suggest that complementarity between the Gag lattice and the Env-CT is a critical factor in Env incorporation and supports previous studies implicating residues in the Gag matrix domain (Gag-MA) for mediating Env acquisition^14,15^.

Interrogating the nanoscale diffusivity of single Env trimers on the cell surface was critical to confirm that removal of the Env-CT leads to increased nanoscale mobility of Env trimers in the presence of active HIV-1 assembly sites. We demonstrate using single-particle tracking of Env that, indeed, CTΔ144-Env trimers are far less confined/immobilized relative to WT-Env, on the tens of nanometers resolution scale, and measured over a large population. Furthermore, Env trimers possessing the *d8* mutation, unable to achieve high-angular distributions in the Gag lattice of budding particles on COS7 cells, showed a marked increase in population mobility relative to WT-Env, supporting the hypothesis that complementarity between the Env-CT and Gag lattice is critical for trapping and particle incorporation.

An alternative interpretation of the high-angular distributions achieved by CTΔ144-Env mutants could also be explained by the presence of a larger quantity of Env on the plasma membrane (Fig. 4b). Within this interpretive framework, the higher plasma membrane CTΔ144-Env trimer density would enable statistical sampling of the Gag lattice during the lifetime of assembly and achieve higher angular distributions on budding particles. We feel that this scenario is not plausible, however, because it does not explain the potent particle incorporation defect observed with CTΔ144-Env, despite higher cell surface densities. In turn, the increased mobile fraction of CTΔ144-Env cannot be explained by saturation of the finite number of Gag lattices on the cell surface because previous studies have shown that CTΔ144-Env incorporation is nonsaturable over a wide range of surface densities, unlike WT-Env^16^. We suggest instead that increased mobility through the Gag lattice reduces the likelihood of Env being present as a particle is abscised from the cell. Collectively, our data support previous interpretations of the role of the Env-CT in regulating Gag lattice incorporation and, through direct visualization and quantification, enables a nanoscale perspective of the encounter between these two viral molecules.

**Fig. 6:**
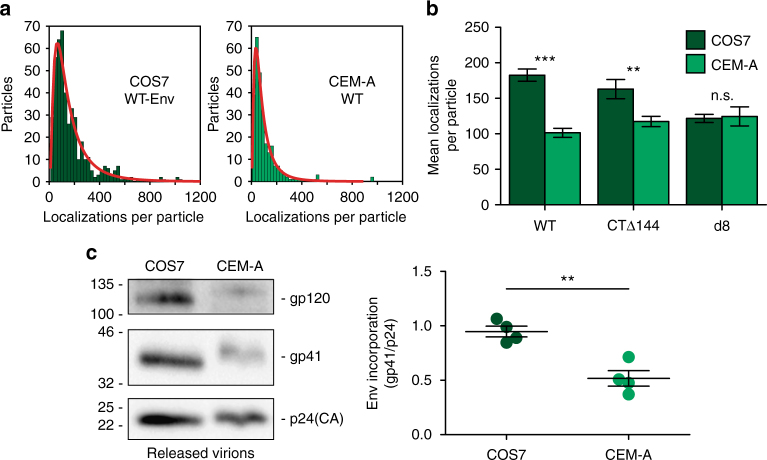
Differential Env incorporation between producer cell types suggests that intracellular retention acts to regulate virus association. **a** Qualitative assessment of the mean number of Env trimers per cell-associated Gag cluster was quantified by counting the number of Env superresolution localizations. Histograms were generated and fit to a lognormal distribution to quantify the mean and standard deviation of the number of Env localizations within each segmented assembly site volume. It is important to note that localization count is not a direct quantification of molecule count due to the variable number of fluorophores per antibody, the unknown labeling stoichiometry per Env trimer, and the repeated appearance of a single fluorophore during a single superresolution experiment. As the aforementioned factors are consistent for each experimental condition, localization counts will be generally proportional to molecule counts. **b** The parameters of the lognormal fits of each distribution were used to compare general levels of Env incorporation between producer cell types for each Env genotype. Bars represent means and s.e.m. of Env channel localizations per particle (WT COS7 = 182.6 ± 8.6; WT CEM-A = 101.5 ± 6.3; *CTΔ144* COS7 = 163.1 ± 13.5; *CTΔ144* CEM-A = 117.4 ± 7.2; *d8* COS7 = 121.7 ± 5.8; *d8* CEM-A = 124.6 ± 13.4). See Supplementary Table 1 for *n* values. ***P < 0.0001, **P < 0.005, n.s. *P* = 0.8445 as assessed by a two-tailed unpaired *t*-test with Welch’s correction. **c** Western blots of released virions were used to assess levels of WT-Env incorporation in virus produced by CEM-A versus COS7 cells. Levels of gp41 (Env) were normalized to the amount of p24 (Gag). Virus produced by COS7 cells displayed a mean ratio of 0.95 ± 0.10 gp41 to p24 (*n* = 4), whereas virus produced by CEM-A cells displayed a mean ratio of 0.52 ± 0.14 gp41 to p24 (*n* = 4). Bar graph on the right represents mean and s.d. **P *<* 0.005 as assessed by a two-tailed unpaired *t*-test

Our investigation into the nanoscale spatiotemporal dynamics of Env assembly suggested that additional mechanisms beyond lattice trapping and plasma membrane diffusion act to regulate the temporal incoherence of Env encounter with the Gag lattice. Specifically, single-molecule tracking could not explain the differences in angular distributions of WT-Env produced in CEM-A versus COS7 cells. We hypothesized that endocytosis and intracellular retention of Env trimers could be utilized by HIV-1 to regulate the timing of Env and Gag encounter in CEM-A cells. We demonstrate that the steady-state levels of intracellular WT-Env were much higher than CTΔ144-Env in CEM-A cells, while intracellular levels of WT- and CTΔ144-Env were very similar in COS7 cells, and surface levels of WT-Env were even slightly higher than CTΔ144-Env. These results suggest that CEM-A cells possess host cell trafficking machinery responsible for interaction with the Env-CT, which leads to intracellular retention in a transferrin-positive compartment (Fig. 4a and Supplementary Fig. 18a), whereas COS7 cells are altered in this trafficking machinery and thus, never significantly sequester a large fraction of intracellular Env. This significantly reduced intracellular retention in COS7 cells, by failing to sequester Env during early Gag lattice assembly, explains the unbiased angular distribution of WT-Env observed on particles produced by COS7 cells, as well as the higher density of Env incorporation per particle (Fig. 6).

Unlike CTΔ144-Env, the d8-Env mutation does not appear to impede specific internalization of Env, and its cell surface density is depleted compared to WT-Env in both cell types. The relatively small percentage of confined/immobilized d8-Env, combined with the high levels of intracellularly retained d8-Env compared with WT-Env, suggests that this Env mutant does not become trapped by Gag lattices and is then largely endocytosed and trafficked to an intracellular compartment. It is possible, however, that d8-Env trimers are also unable to recycle to the plasma membrane upon internalization, which would exacerbate particle incorporation defects and increase the steady-state levels of intracellular d8-Env relative to WT-Env. The loss of cell-type-dependent differences in d8-Env incorporation (Fig. 6b) seems to support a cell-type-independent occlusion of d8-Env from the Gag lattice, irrespective of the apparently cell-type-dependent intracellular retention of WT-Env. When the intracellular pool of d8-Env is photobleached and allowed to recover, a larger fraction of d8-Env recovers relative to WT-Env (63.6 ± 1.1% versus 44.6 ± 3.4%, respectively) despite having a smaller plasma membrane pool (Fig. 4), suggesting that the higher mobile fraction of d8-Env trimers, relative to WT-Env, on the plasma membrane may contribute to this recovery. This result could also be explained by a defect in recycling of d8-Env trimers. It is important to note that these data do not differentiate between flux due to endocytosis and flux due to exocytosis, and thus cannot conclusively prove a defect in d8-Env recycling to the plasma membrane, but these results are consistent with previous studies of related Env-CT mutants^14,27^. This mechanism of exaggerated intracellular retention would explain our observation of an induced neck bias for d8-Env distribution in COS7 cells and is analogous to the intracellular retention of WT-Env and neck bias we observe in CEM-A cells. Further study is warranted to define the exact host cell machinery responsible for intracellular retention of Env and to conclusively determine whether it is specifically impaired by the *d8* mutation. The discovery that the host cell vesicular recycling protein, FIP1C, is responsible for regulating Env particle incorporation^16,27^ suggests that this factor may regulate intracellular retention and temporal modulation of the encounter between Env and Gag. As there are numerous steps in vesicular trafficking and endosomal cargo recycling, it is possible that other cell-type-specific machinery exists to regulate Env intracellular retention.

**Fig. 7:**
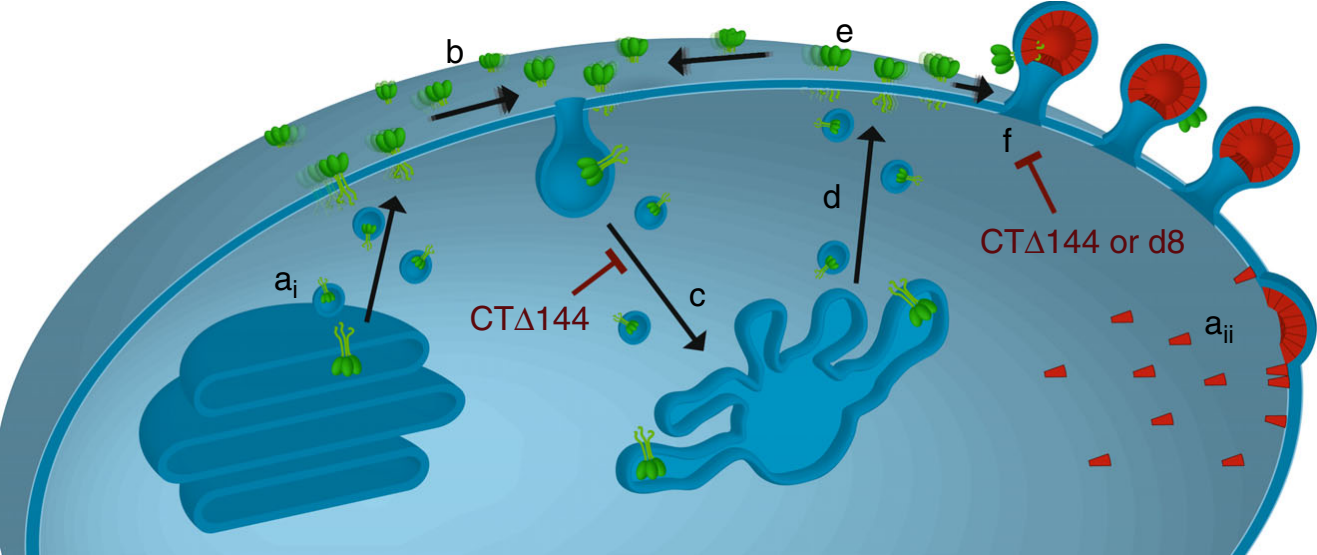
Working model for the fate of single Env trimers during HIV-1 assembly. **a**_i_ Env is synthesized in the endoplasmic reticulum and trimers traffic to the plasma membrane through the canonical secretory pathway. **a**_ii_ Gag is synthesized in the cytoplasm and targets the plasma membrane, then forming a protein lattice and budding from the plasma membrane. **b** Env trimers diffuse laterally on the plasma membrane and either become entrapped in assembling Gag lattices or are rapidly internalized through clathrin-mediated endocytosis. Truncation of the Env-CT (*CTΔ144*) impairs Env internalization, leading to increased levels of surface-exposed Env. **c** Endocytosed Env populations are sorted and trafficked to the recycling endosome. **d** In T cells, Env populations are predominantly retained intracellularly, while limited quantities of Env are recycled back to the plasma membrane. This model suggests that specific recycling is regulated by interactions between host cell factors and elements of the Env-CT, leading to a marked reduction in the levels of surface-exposed Env. **e** Upon recycling of Env back to the plasma membrane, Env trimers diffuse laterally on the plasma membrane and either become entrapped in assembling Gag lattices or are re-endocytosed. **f** Intracellular retention of Env leads to low levels of surface-exposed Env, increasing the probability that Env trimers will encounter pre-formed lattices rather than low-order Gag oligomers, resulting in the observed neck-biased distributions. Truncation of the Env-CT results in a defect in Env’s ability to interact with and become sterically trapped by Gag lattices. The *d8* mutation disrupts complementary interactions between the Gag lattice and the Env-CT resulting in exclusion of d8-Env trimers from the lattice. This exclusion will result in re-endocytosis of Env trimers and potentiate intracellular accumulation. These disruptions to the HIV-1 assembly process ultimately manifest as bulk defects in Env trimer incorporation into released particles

Collectively, this study supports a model wherein rapid Env-CT-dependent internalization and sequestration of Env in endocytic compartments, followed by limited recycling of Env back to the plasma membrane, restricts extracellular display and delays the encounter of Env with budding Gag lattices until late in the assembly process. This mechanism sterically consigns the long Env-CT to the peripheries of Gag lattices and the necks of budding particles, limiting the density of Env incorporation to individual virions (Fig. 7). Importantly, this trafficking itinerary for Env trimers appears to be cell-type-specific, as angular Env distributions were unbiased when expressed in fibroblast-like cells. We propose that the combination of host cell trafficking factors and Gag lattice complementarity are crucial for maintaining steady-state distributions between internally retained and virus-associated Env. These findings have significant implications for understanding the mechanisms of HIV-1 immune evasion and point to a tightly regulated assembly pathway limiting cell surface exposure of HIV-1 Env. While limiting the incorporation of Env trimers into virus particles reduces viral infectivity^28^, this mechanism potentially contributes to the lack of antibody diversity generated by an infected host and reduced intensity of antibody-dependent cell-mediated cytotoxicity during HIV-1 infection^29^.

## Methods

### Antibodies

Anti-Env antibodies b12^30–33^ and 2G12^34,35^ were acquired from the NIH AIDS Reagent Program (Germantown, MD) or purchased from Polymun (Klosterneuburg, Austria), respectively (the b12 reagent was obtained through the NIH AIDS Reagent Program, Division of AIDS, NIAID, NIH: anti-HIV-1 gp120 monoclonal (IgG1 b12) from Dr. Dennis Burton and Dr. Carlos Barbas). Anti-FLAG clone M2 antibody was purchased from Sigma-Aldrich (#F3165). Anti-capsid (Gag-CA) KC57 antibody was purchased from Beckman Coulter (Brea, CA). All anti-Env (b12 and 2G12) antibodies were coupled directly to AlexaFluor 647 *N*-succinimidyl ester (#A37566; Life Technologies). The anti-FLAG M2 and anti-CA KC57 antibodies were coupled directly to AlexaFluor750 *N*-succinimidyl ester (#A20111; Life Technologies). Typically, a labeling ratio 1.8–2.8 AlexaFluor dyes per IgG molecule was achieved. Anti-Env 2G12 antibody was used as primary and goat anti-human IgG with 6 nm Au (Electron Microscopy Sciences, Hatfield, PA) was used for immunogold labeling.

### Recombinant antibody fragment production

The anti-Env b12 Fab fragment recombinant expression vector was a kind gift from Dr. Dennis Burton^30^. The pCOMB3H-b12 expression vector was deleted for the *pIII* gene by digestion with SpeI and NheI (New England Biolabs; Ipswich, MA) and ligation of the vector backbone. Expression of b12 was carried out in *Escherichia coli* XL1 Blue competent cells (Stratagene; San Diego, CA) as previously described^36^. To purify b12, bacterial cell pellets were resuspended in PBS pH 7.4 containing a final concentration of 0.2 mM PMSF (#P-470-10; Gold Biotechnology, Inc.; St. Louis, MO) and sonicated to produce cellular lysate. Clarified cell lysates were purified by protein G affinity chromatography (#P-430–5; Gold Biotechnology, Inc.; St. Louis, MO). Eluates were pooled and dialyzed overnight in PBS, pH 7.4 at 4 °C. The b12 Fab, typically 99% pure, was conjugated with Atto565 *N*-succinimidyl ester (#72464; Sigma-Aldrich; St. Louis, MO). Typically, a labeling ratio of 1 Atto565 dye per b12 Fab molecule was achieved. This monovalent reagent was capable of labeling HIV-1 Env expressing cells without the concern of receptor crosslinking, unlike bivalency crosslinking in the case of full IgG molecules.

### Cell lines

The COS7 parental cell line was obtained from ATCC (CRL-1651; Manassas, VA). The HEK293T cell line was also obtained from ATCC (CRL-11268). The CEM-A cell line was obtained through the NIH AIDS Reagent Program, Division of AIDS, NIAID, NIH: CEM-A from Dr. Mark Wainberg and Dr. James McMahon, CEM-CL10^17^. Cells were maintained at 37 °C with 5% CO_2_.

### Growth medium

Complete growth medium was prepared with 10% fetal bovine serum (#35-011-CV), 2 mM L-glutamine (#25-005-CI), and 1× penicillin-streptomycin (#30-002-CI) in DMEM (#17-205-CV) for the COS7 cell line or in RPMI (#17-105-CV) for the CEM-A cell line (Corning; Herndon, VA). The CEM-A cell line growth medium was additionally supplemented with 1% hypoxanthine, thymidine (HT) solution (25-047-CI; Corning).

### Production of replication and release defective HIV-1

The modified NL4–3 reference genome was cloned into an SV40 ori-containing backbone (pN1 vector; Clontech/Takara Bio USA, Mountain View, CA) and was used as a transfer plasmid. Briefly, the following modifications were made to the NL4–3 reference genome for the iPALM experimental imaging approach: (i) addition of a coding C-terminal FLAG tag to the *gag* open reading frame (GSDPSSQ_500_-SGDYKDDDDK*; referred to as Gag in the manuscript)^22^, (ii) mutation of the p6 PTAP motif (_455_PTAP_458_-LIRL)^18^, (iii) deletion of *pol* by removal of the BclI-NsiI fragment in this open reading frame, and (iv) removal of the 5′ portion of the *nef* open reading frame and replacement with a probe containing the *photoswitchable cyan fluorescent protein 2* (PSCFP2) open reading frame fused to a myristoylation coding sequence^23^. Mutational modifications to the Env cytoplasmic tail were created on the aforementioned genetic background: WT- *wild-type env*, CTdel144 - ΔCT removal of the last 144 amino acids of the Env cytoplasmic tail by premature stop codon^5^, or *d8* - deletion of 5 amino acids (802–806, numbering references NL4–3 gp160) from the second predicted alpha helical domain of the Env-CT, LLP-3^14^.

Single-round infectious viruses were produced by transfecting HEK293T cells with the psPAX2 packaging vector (a gift from Didier Trono, Addgene plasmid #12260) and pseudotyped with pVSV-G (gift from Dr. Xuedong Liu, University of Colorado, Boulder). HEK293T producer cells were allowed to express for ~48 h before virus was collected, passed through a 0.45 µm filter, and frozen at −80 °C.

### Production of infectious viruses and biochemical analysis

Infectious viruses were produced by transfecting HEK293T cells with pNL4-3 WT-, CTdel144-, or d8-Env and pseudotyped with pVSV-G. HEK293T producer cells were allowed to express for ~48 h before virus was collected, passed through a 0.45 µm filter, and frozen at −80 ^o^C. To quantitatively assess Env incorporation and particle production biochemically (Fig. 6 and Supplementary Fig. 16), single-round infection was performed on COS7 and CEM-A cells using ~1.5–4 RT-CPM/µL of virus. Approximately 40 h post infection, virus was collected, passed through a 0.45 µm filter, layered on a 20% (w/v) sucrose cushion, and concentrated at 20,000 × *g* for 2 h at 4 °C. Env gp41 and gp160 were detected with anti-Env 10E8^37^ at 0.5 mg per mL, anti-Env 16H_3_ 0.2 mg per mL, and p24(CA) and Pr55Gag were detected with HIV-IgG at 10 mg per mL (the following reagent was obtained through the NIH AIDS Reagent Program, Division of AIDS, NIAID, NIH; anti-HIV-1 gp41 monoclonal (10E8), from Dr. Mark Conners and anti-HIV-1 Env 16H3 monoclonal antibody from Drs Barton F. Haynes and Hua-Xin Liao^38^). Secondary detection of anti-HIV antibodies was performed with anti-mouse HRP (16H3) and sheep anti-human HRP (HIV-IgG and 10E8). Full western blots are shown in Supplementary Fig. 19.

### Interferometric photoactivation localization microscopy

Instrumentation associated with the iPALM method is described by Shtengel et al.^21^, but with the *z*-axis measurement range extended to 750 nm with phase unwrapping using astigmatism^39^. Images were collected in frame transfer mode using three iXon DU-897W EM-CCD cameras (Andor Technology; Belfast, Northern Ireland). An additional 300 mW 750 nm fiber-coupled diode laser (Edmund Optics; Barrington, NJ) with custom collimation optics was added to the microscope setup for imaging of AlexFluor750 (Life Technologies, Carlsbad, CA). The 750 nm laser line was filtered with a 740/35 band-pass filter and the fluorescence emission in the 750 nm channel was filtered through a 795/50 emission filter (Chroma Technology; Bellows Falls, VT). Filters used for the 488 nm and 647 nm channels were as described previously^22^.

### Transmission electron microscopy

Ultra-thin sections (60 nm) were cut on a Reichert Ultracut S from a small trapezoid positioned over the tissue and were picked up on copper mesh grids (EMS). Sections were imaged on a FEI Technai G2 transmission electron microscope (Hillsboro, OR) with an AMT digital camera (Woburn, MA).

### Spinning disk confocal microscopy

Imaging was performed with a customized inverted Nikon Ti-E microscope (Solamere Technology Group Inc., Salt Lake City, UT) using a 60×, CFI Plan Apo Lambda 1.4 NA oil-immersion objective (Nikon Instruments; Melville, NY). Three fiber-coupled 488 nm, 561 nm, and 640 nm lasers (OBIS CW solid-state lasers, Coherent; Santa Clara, CA) were used in combination with a CSU-X A1 spinning disk unit (Yokogawa Electronics; Tokyo, Japan) to excite and collect confocal sections of AlexaFluor488, Atto565, or AlexaFluor 647 fluorescence, respectively. Laser powers were measured at the sample stage. For FRAP experiments, the 561 nm laser was operated at ~340 ± 5 µW cm^−2^. For fixed cell experiments (see surface stain and internalization assays), the 561 nm laser was operated at ~200 ± 5 µW cm^−2^. The 488 nm laser was measured at ~12 ± 1 µW cm^−2^, and the 640 nm laser was measured at ~315 ± 5 µW cm^−2^. A quad-pass dichroic mirror (Chroma Technology) was used to reflect the excitation sources and transmitted fluorescence was filtered using either a 525/50 band-pass (ET525/50 m), 570 long-pass (ET570lp) emission filter for AlexFluor488 and Atto565 imaging, respectively (Chroma Technology). For imaging of AlexaFluor 647 in separate experiments, a 700/45 band-pass filter was used (T660lpxr; Chroma Technology). Fluorescence emission was detected using an ImagEM X2 electron multiplying charged-coupled device (EM-CCD, C9100-23B; Hamamatsu, Hamamatsu City, Japan). Rapid point photobleaching was performed on specimens using a rear-port coupled *i*Las^2^ laser illuminator (BioVision Technologies, Exton, PA) coupled with a 100 mW 405 nm OBIS laser (Coherent; Santa Clara, CA). A 405 nm reflecting dichroic and long-pass transmitting broadband dichroic mirror was used for laser coupling into the rear-aperture of the objective (ZT405rdc; Chroma Technology).

### Cell preparation for interferometric-PALM measurements

Bare 25 × 45 nm Au nanorods (#A12-25-700, Nanopartz, Loveland, CO) were used as fiducial markers, and were deposited onto 25 mm #1.5 coverslips (Warner Instruments, Hamden, CT). A 20–50 nm layer of SiO_2_ was then sputtered over the gold-bearing coverslips (Denton Explorer sputtering system; Denton Vacuum, Moorestown, NJ). Coverslips were cleaned for single-molecule imaging as described^40^. After air drying, coverslips were placed in 3.5 cm culture dishes and coated with fibronectin in PBS (#FC010; Millipore, Billerica, MA). COS7 or CEM-A cells were seeded onto Au-, SiO_2_-treated coverslips with ~2–8 × 10^5^ cells in complete growth medium. Cells were typically infected immediately after plating with VSV-G pseudotyped NL4–3 virus of various genotypes and allowed to express viral proteins for 36–42 h. For iPALM measurements, cells were then fixed with 4% paraformaldehyde (#15710) and 0.2% glutaraldehyde (#16220) for 30 min (Electron Microscopy Science). The fixing solution was quenched with successive washes in 30 mM glycine in PBS solution (#G8898; Sigma-Aldrich). Cells were permeabilized with 0.2% Triton X-100 (Sigma-Aldrich), blocked in 10% BSA in PBS, and stained for 12 min with custom-made antibodies (KC57 AF750 at ~400–900 ng per mL, αFLAG AF750 at ~900 ng per mL, 2G12 AF647 at ~200 ng per mL, and b12 AF647 at ~200 ng per mL) diluted in 6% BSA/PBS (#A7906; Sigma-Aldrich). Samples were then washed thoroughly with PBS solution containing 0.2% BSA and 0.05% Triton X-100 (T8787; Sigma-Aldrich). The coverslips were rinsed and overlaid with 150 µL of dSTORM switching buffer (10% glucose, 70 µg mL^−1^ glucose oxidase, 13 µg mL^−1^ catalase, 50 mM Tris, and 25 mM NaCl)^41^. An 18 mm #1.5 coverslip, cleaned as described above, was attached over the cells and sealed with epoxy (Elmer’s Products, Inc; Columbus, OH) and vaseline (Unilever; Greenwich, CT). Cell specimens were imaged immediately upon sealing.

Typically, 40,000–60,000 frames were collected for each channel using frame transfer mode on the EM-CCD. The 488 nm channel (PSCFP2 excitation) was ~0.4 kW cm^−2^, 647 nm channel (AlexaFluor 647 excitation) was ~3 kW cm^−2^, and 750 nm channel (AlexaFluor 750 excitation) was ~2 kW cm^−2^. Exposure times were 50 ms, 20–30 ms, and 50–75 ms for the 488 nm, 647 nm, and 750 nm channels, respectively. Three-channel iPALM image registration was performed using Au nanoparticles with extended fluorescence observation under 488, 647, and 750 nm illumination, which allowed for registration of the three channels as described.

### Spinning disk confocal imaging and FRAP rate measurements

For surface staining assays, 18 mm coverslips were treated with fibronectin in PBS and seeded with COS7 or CEM-A cells. Cells were immediately infected with virus as described above and allowed to express for 40 h. Samples were then fixed with 4% paraformaldehyde and 0.2% glutaraldehyde for 30 min and quenched with 30 mM glycine solution as described above. Cells were then blocked for 30 min in 10% BSA in PBS and stained for 12 min with custom antibodies against Env (2G12 AF647 at ~600 ng per mL, b12 AF647 at ~600 ng per mL) in 6% BSA. Samples were thoroughly washed with 0.2% BSA in PBS, then mounted in Fluoromount-G (#0100-01, Southern Biotech), placed on a clean coverglass and sealed. Oversampled Z-stacks of 300 nm slices were collected using the 640 nm laser line at 100 ms exposure times. Cells were segmented along the cell peripheries by thresholding of maximum projections of each stack. The cell periphery mask was then used on integrated Z-projection images (2D) for the same cell to compute the integrated intensity of each cell. This integrated intensity (AU) was then normalized to the segmented cell area.

For analysis of intracellular Env pools, 18 mm coverslips were likewise treated with fibronectin in PBS and seeded with COS7 or CEM-A cells. Cells were infected as above and allowed to express for 40 h prior to pulse-chase labeling. Cells were blocked with 10% BSA in complete media for 30 min at 37 °C, then pulse-chase labeled with custom anti-Env b12-Fab fragments conjugated to Atto565 (b12-Fab Atto656 at ~60 nM) and transferrin conjugated with AlexaFluor488 (Transferrin-AF488 at ~25 µg per mL, #T13342; Thermo Fisher) for 12 min at room temperature. Cellular specimens were successively washed thrice with complete media, fixed in 4% paraformaldehyde and 0.2% glutaraldehyde for 30 min, and quenched in glycine as above (~15 min post-staining). Samples were then mounted in Fluoromount-G. Images were acquired of equatorial sections of cells, where intracellular compartments were in optimal focus, as assessed by Transferrin-AF488 labeling. Three image regions were manually segmented using ImageJ (NIH) for each cell to assess the degree of internalization/retention of Env for each genotype: (i) the outer edge of each cell, (ii) the inner edge of the equatorial section of the plasma membrane, and (iii) the intracellular pool which correlated with staining in the Transferrin-AF488 channel. The total intensity of intracellular regions was normalized to the integrated fluorescence intensity of both intracellular and plasma membrane regions of interest.

For FRAP assays, CEM-A cells were seeded to 25 mm coverslips and infected for 40 h. Live cell pulse-chase labeling of surface-exposed Env was performed as described above, though in the absence of Transferrin-AF488. Focus was set at equatorial sections of cells as assessed by the highest intracellular Env signal. Time-lapse image series were acquired at 1 min intervals for each FRAP experiment. Five intervals were acquired prior to photobleaching to establish an initial baseline and steady-state signal for the intracellular compartments. A region of interest around the intracellular compartment was photobleached using the *i*Las^2^ photobleaching galvo scanner and 405 nm laser light for 25 s immediately before the sixth time series was acquired. Recovery of the intracellular pool was assessed for 35 further 1 min interval frames.

For FRAP analysis, the background-corrected integrated intensity for the region of interest (ROI) was measured before and after photobleaching. The rate of photobleaching from image sampling (not due to ROI point bleaching) was assessed by measuring the intensity of peripheral control cells through each frame interval and was found to be linear over the time series. The background-corrected integrated FRAP ROI was corrected for photobleaching using a linear model of fluorescence decay. The percent recovery in FRAP regions of interest was normalized for each experiment to allow the first frame after photobleaching to have a relative intensity of zero percent. After recovery normalization, biological replicates were then averaged for each genotype. The standard deviation of intensity recovery was used to assess the error in this measurement across all biological replicates. The rate constants for exponential signal recovery in each genotype were extracted from the averaged recovery traces that were fit to an exponential function. The standard deviations in each time point were normalized according to the inverse of the largest error in the time series and used as weights for the exponential fitting function.

### Immunogold labeling and transmission electron microscopy

CEM-A cells were infected with attenuated single-round virus for the appropriate NL4–3 genotype and allowed to express viral proteins for ~40 h, as described above. Cells were stripped from dishes with cell stripper (#25-056-CI, Corning) and then fixed in suspension in 0.2% glutaraldehyde and 4% paraformaldehyde. The fixed cell suspensions were blocked in 10% BSA/PBS and then Env was labeled simultaneously with anti-Env 2G12 (~600 ng per mL) and anti-Env b12 IgG (~600 ng per mL) in 6% BSA solution for 30 min. Cell samples were pelleted and resuspended thrice in 0.2% BSA and then labeled with goat anti-human IgG conjugated to 6 nm gold (#25203; Electron Microscopy Sciences) diluted 1:5 in 6% BSA for 2 h. Cells were then pelleted and resuspended four times in PBS containing 0.2% BSA. After the final wash, cells were pelleted and post fixed in 2% glutaraldehyde/PBS. The pellets were resuspended in 5% agarose that was allowed to harden and then cut into small pieces for processing. Cells were first rinsed three times in 0.1 M sodium cacodylate buffer (pH 7.4), then post fixed in 2% osmium tetroxide for 1.5 h. After three washes in water, the cells were dehydrated through a graded acetone series (3× 25%, 50%, 75%, 90%, 95%, 100%) for 10 min each, and infiltrated with LX112 resin. The cells were embedded and cured for 48 h at 60° C in an oven. Ultra-sectioned samples were cut as described above (60 nm) and imaged on copper mesh grids.

Neck lengths of budding particles were estimated using ImageJ (NIH). Those budding particles that were sectioned evenly about the transverse profile were used for analysis. Briefly, the particle centroid was estimated from the intersection of two orthogonal diameter estimates. The local membrane plane was estimated by a line orthogonal to the budding axis and lying on the center of the membrane electron density.

### Single-molecule tracking

The described iPALM instrument was used to perform single-molecule imaging of Env diffusion using a single objective and camera operating in total internal reflection fluorescence mode. CEM-A cells plated onto clean 25 mm coverslips were infected with attenuated single-round virus for the appropriate NL4–3 genotype and allowed to express viral proteins for 40 h as described above before performing pulse-chase labeling of surface-exposed Env. COS7 cells plated to cleaned, fibronectin-coated 25 mm coverslips were transiently transfected with pSVNL4-3 vectors lacking *pol, vif, vpr, nef*, and the 3′LTR. An additional pSVNL4-3 vector with an identical genotype, but containing an internal GFP tag between the MA and CA domains^22^, was used to visualize the presence of HIV-1 assembly sites. Importantly, the internal GFP tag expression vector was used at a ratio 1:5, tagged to untagged Gag, to prevent assembly defects. Transiently transfected cells were allowed to express for ~18–20 h before pulse-chase labeling of surface-exposed Env was performed.

Cellular expression of Env was detected by staining with custom anti-Env b12 Fab fragments conjugated to the Atto565 dye (~10 nM; Atto-tech, Siegen, Germany) at 25 °C for 12 min in 6% BSA and complete media. Cellular specimens were then successively washed thrice with complete media prior to imaging at 25 °C. Cells displaying a relatively low, but uniform density of Gag clusters were selected for each imaging experiment to ensure large Gag patches did not significantly interfere with the measurement of the mobile fractions of Env. The total kinetic cycle time per frame for each experiment ranged from 17 to 19 ms and the camera was operated in frame transfer mode.

### Superresolution image analysis

iPALM superresolution images were generated as previously described^21,22^. Raw localizations in each data set were filtered by positional uncertainty (*σ*_*x*,*y*,*z*_) to less than 40 nm in the *X*-, *Y*-dimensions and 30 nm in the *Z*-dimension (Supplementary Fig. 1) and all three channels were aligned by transform of broadly fluorescent gold fiducial markers (Supplementary Fig. 2). Segmentation of individual clusters of localization in the Gag and Env channel were performed as previously described^22^. Typical regions of interest were ~1.5–2.5× the 2D area of each HIV-1 Gag cluster depending on the HIV-1 Gag cluster density in the cell. A custom written Matlab program (Mathworks, Natick, MA) then extracted the segmented localization coordinates and uncertainties for all three channels (750, Gag; 647, Env; and 488, membrane) along the entire *Z*-depth. Each HIV Gag cluster was then inspected for collisions with other HIV-1 Gag clusters, circularity, presence of Env, and definability of the local membrane topology. The Gag localization cluster was least-squares fit to a spherical shell model for reference alignment. Any residual ghost localizations which were improperly reassigned during the phase unwrapping algorithm were computationally reassigned using a peak finding algorithm along the optical *Z*-axis of the segmented volume in each of the channels. Only fractional clusters of peaks which fall exactly one fringe order away (±full-width at half-maximum) of the *Z*-peak density were reassigned to the real cluster of localizations. All segmented volumes were manually inspected for proper reassignment of ghost peaks in each channel. Ghost localizations which were not identified to be part of a fringe were discarded from the segmented volumes. All cell type and genotype volumes were treated identically for these segmentation routines. After all inspection and ghost reassignments, this typically resulted in removal of 15–50% of segmented assembly sites for each imaged cellular specimen. Those segmented assembly sites which satisfied the filtering criteria were then converted to probability densities and aligned via the Gag shell fit centers as described previously^22^. The standard error in the radius parameter was typically less than 20 nm for each Gag cluster and followed an approximately normal distribution (Supplementary Fig. 3). The standard deviation of all standard error measurements calculated from the fit radius was no greater than 5 nm for each Env-CT genotype and cell-type tested.

### Filtering assembly sites

Those segmented virus assembly sites which performed poorly in the Gag centroid and shell fitting algorithm due to a lack of single-molecule localizations describing the Gag volume were discarded. Virus assembly sites which lacked sufficient local membrane sampling (<50 localizations per segmented area) were filtered from the total pool in each cell and virus genotype. This discarded pool typically consisted of less than 5% of the segmented data.

### Rotational alignment by local membrane plane fitting

To enable angular probability density measurements of Env distributions on averaged single HIV-1 assembly sites, we performed weighted principle component analysis (PCA) using the Matlab function: pca with the default singular value decomposition algorithm option and centration of the data. The localization uncertainty in all three dimensions, *σ*_localization,*x*,*y*,*z*_, for single virus assembly sites was averaged and normalized to one. The normalized error was then used as weights for the PCA function. The normal vector for the plane was extracted from the principle component coefficients and the residual error was determined from computing the real-space distances of each localization centroid to the fit plane. These values were typically below 5 nm for each dimension. The angular deviations of the computed normal vectors were used to perform a rigid affine transform of the aligned Gag shell (AF750 channel), the corresponding Env signal (AF647 channel) and the corresponding membrane plane (PSCFP2 channel). All channels were aligned to the real-space optical axis (*Z*) using the weighted PCA estimated normal vectors representing the virus bud polarity.

### Viral bud neck normalization

We observed a normally distributed range of neck lengths for each viral bud, estimated from the centroid of the Gag shell and the centroid of the membrane plane after alignment with the optical axis (150 ± 92 nm; see Supplementary Fig. 6). Those Gag centroids residing too close to the membrane planes, representing early stages of Gag-induced membrane curvature, were discarded (typically <1.25 × *σ* from the mean neck length). Typically, however, the flat lattices of Gag, prior to induction of membrane curvature, were discarded in early stages of processing due to poor fitting to the Gag shell model. Those few Gag assembly sites whose centroids resided at a distance larger than 300 nm from the membrane centroid, likely representing sheared or shed particles, were observed infrequently and represented less than 6% of all assembly site measurements. Importantly, the distribution of neck lengths we observed were in good agreement with estimated neck lengths observed by thin-section transmission electron microscopy (127 ± 38 nm from the proximal membrane plane; Supplementary Fig 15e).

For purposes of visualizing the Gag and Env probability densities (see Fig. 2a and Supplementary Fig. 13), we performed distance normalization based on the mean neck length measured in each data set. Importantly, normalization of the neck lengths was only performed for visualization purposes. However, the absolute axial distance of the membrane plane from the Gag centroid has no effect on determination of the fitted plane normal vectors, and thus, will not affect the aligned angular probability of Env about the Gag lattice.

### Single cluster averaging

For translationally and rotationally aligned single virus assembly sites, the centroid coordinates and localization uncertainties for each channel (Gag, Env, and membrane) were converted into 3D probability densities using 1 nm voxels. This volumization algorithm was parallelized using CPU multithreading and the Matlab parallel-processing toolbox. The pre-aligned probability volumes were then integrated to calculate the total probability over each genotype or cell-type measurement of virus assembly sites. The final probability densities were then trimmed into cubes of 601 × 601 × 601 nm with the center voxel (at 301 × 301 × 301 nm) representing the centroid of the Gag shell.

### Probability density analysis

Integrated probability densities for the aligned HIV-1 Env channel were used to determine the angular probability distributions for each Env genotype and for each cell type. Using the center voxel for each volume, a custom 3D angular averaging algorithm was used. Briefly, the algorithm integrates along a cone described by each azimuthal (elevation) angle, *φ*, on the interval −90°:1°:90°, where the elevation angle from −90° to 0° represents the southern hemisphere (neck) of the volume and 0°–90° represents the northern hemisphere (crown) of the volume. The probability density for each elevation angle was integrated along the length of a vector originating at the center voxel with a radial length of 300 nm. This vector was used to integrate about the polar angle, *θ*, to compute the probability density of the cone described for each azimuthal angle, *φ*.

In order to determine the angular resolution for the angle *φ* using this method (and thus the appropriate bin size for the angular histograms), we generated random pairs of half data sets for each Env genotype and the producing cell type, computed the angular (*φ*) probability of Env about the Gag shell for each half data set, and then phase correlated the one-dimensional angular probability vectors of the two half data sets (see Supplementary Fig. 12). Briefly, the coordinates for individual virus assembly sites were randomly divided in half. The half data sets were then aligned, rotated, and volumized to create two probability density volumes for each genotype or cell type. The half data set volumes for each data set were then passed to the 3D averaging routine described in the previous paragraph. These two resulting integrated angular (*φ*) probability vectors were then cross-correlated using the Matlab function xcorr. The lag at which correlation was maximum between the half data set vectors was used as a measure of the sampling density for the full data set. The lags were never larger than 20° with an average of 9° between any random half data set for all Env genotypes and producing cell types tested. This result indicates sufficient sampling of Env at HIV-1 assembly sites to conclude fine spatial differences in probability density under specific biological perturbations (genotypic or cell-type changes).

### Visualization of probability densities

The 3D matrices of aligned and averaged virus assembly sites were isosurfaced using the indicated threshold (see individual figures) using Matlab. Isosurfaced probability densities were isocapped on the sliced open face of the shell using an identical threshold value. To enable direct comparison for three-channel probability densities, each subfigure was rendered using an identical isosurface and isocap threshold for the respective channels.

### Simulation of angularly biased Env clusters

We developed a particle simulation algorithm, which utilized seed parameters closely matching the geometric parameters of HIV-1 assembly sites, localization densities, estimated re-appearance (blinking) parameters, and localization uncertainty values for AF647-labeled Env, AF750-labeled Gag, and the S15-PSCFP2 membrane probe.

### Monte Carlo simulation of errors in angular measurement

To assess the overall accuracy measurement of the angular probability distribution of Env from aligned multicolor 3D superresolution imaging data sets, we simulated the Gag shell, local membrane plane, and Env localization centroids residing precisely at the equator (*φ* = 0°). The Env centroid and localization uncertainty were modeled as a Dirac *δ* function (*σ*_*φ*_ → 0°) to assess the effects of both Gag shell fitting and uncertainties in the principle component analysis of the approximated membrane plane. The localization precisions in the *x*, *y*, and *z*-dimension for all simulated channels were selected pseudorandomly from a normal distribution centered at the estimated mean localization precisions measured for each fluorescent probe averaged over all data sets (Supplementary Fig. 1). We additionally simulated the small registration errors between the three superresolution channels, by randomly displacing the simulation structures from one another along a random vector distributed about the mean *σ*_registration(*x*,*y*,*z*)_ averaged over all data sets (1):

1 $$\Delta _{{\mathrm{centroids}}\;(\mathrm x,y,z)} = \sqrt {\sigma^2_{{blue,green}} + \sigma^2_{{red,green}}}$$

To simulate the uncertainty in the fit radius, localizations for the red Gag channel were first expanded pseudorandomly on a sphere with a radius estimated from our measured mean cluster radius, in good agreement with the actual shell radius of HIV-1 Gag. All Gag localizations were then pseudorandomly displaced from the sphere surface using a lognormal distribution estimated from the mean standard error measurement of all Gag shell fits (σ_shell fitting_ = 13.94 nm). The number of localizations per Gag cluster was pseudorandomly selected from the mean and standard deviation of the empirically derived number of Gag localizations per particle (mean = 147.65 ± 6.90 localizations per cluster; *n* = 2183 clusters). To simulate the uncertainty in the principle component analysis of the membrane plane angles, we modeled the individual membrane planes as Gaussian point cloud discs with *σ*_*x*,*y*,*z*_ estimated from the empirically measured mean of the standard deviations of localization positions for all individual single HIV-1 assembly sites in this study (mean *σ*_*x*/*y*_ = 76.91 nm and *σ*_*z*_ = 29.72 nm; see Supplementary Fig. 5). The randomized localization centroids were bounded in the *X*- and *Y*-dimensions at an area of 4.0 × 10^4^ nm^2^ (200 × 200 nm) to simulate the boxcar segmentation of individual assembly sites adjacent to one another. We used the empirically measured mean localizations per plane (257 ± 11 localizations; s.e.m., *n* = 1980 planes) for pseudorandomly seeding localizations into each plane. Env localizations and membrane plane point clouds were affine transformed about the Gag cluster center pseudorandomly to simulate alterations in the virus bud polarity axis relative to the microscope reference axis. Membrane planes and the single Env localization were displaced pseudorandomly from the Gag centroid, along the simulated budding axis, to mimic the lognormal distribution of bud neck lengths observed with empirically derived data (Supplementary Fig. 6; 127 ± 38 nm from the centroid of the membrane plane to the centroid of the Gag cluster). Simulated HIV-1 assembly sites with a fixed Env angular orientation (Dirac function), which were pseudorandomly modified to incorporate the stochastic parameters described above, were then processed (identical to empirically derived data) using our particle processing software.

Additional synthetic data sets were constrained for the placement of single Env clusters at defined angular intervals (*φ*) to mimic the occurrence of Env angular distribution bias about the budding Gag particle. Data sets biasing Env to the crown of the particle (*φ* = 0 − +90°), the neck of the particle (*φ* = −90 − 0°), or a random distribution about the particle (*φ* = −90 − +90°) were generated. The probability density upon single virus averaging faithfully recapitulates the angular bias constraints from each data set as assessed by integrated angular densities (see Supplementary Fig. 7).

### Individual envelope cluster measurements

As an orthogonal measure of the angular distributions of Env clusters measured by iPALM for different Env genotypes or virus-producing cell types, a 3D cluster segmentation algorithm was designed to quantify Env cluster distributions at single HIV-1 assembly sites (Gag clusters). Briefly, from each data set, the set of Env localizations from a single segmented HIV-1 assembly site was converted into a 3D probability density. This routine, as described above, was parallelized to enable facile throughput for probability density calculations of hundreds of Env clusters using CPU multithreading and the Matlab parallel-processing toolbox. A template matching 3D convolution algorithm was implemented in the Fourier domain to find Env clusters. Manual inspection of over 400 segmented Env clusters, which contained a minimum of 15 localizations and were within 200 nm of a Gag cluster centroid, were used to assess their size distributions (see Supplementary Fig. 8). We found that Env trimer signals were normally distributed and clustered with a tight (less than <25 nm), isotropic *σ* distribution. For each cluster finding routine performed on a single assembly site, template clusters (probability densities) were generated in triplicate from a normally distributed random distribution approximating the size of manually measured Env clusters (*σ* = 7–25 nm). Three-dimensional convolution was performed thrice with each of the three template clusters for a total of nine convolutions on each assembly site probability density. The resulting convolved probability densities were filtered for noise using a singleton voxel filter, thresholded by 3× *σ* of all voxel values, binarized with voxels greater than zero probability, and then regions were linked using a watershed filter (six-connected voxel neighborhood). The centroids of the resulting region objects were then used to calculate the nearest centroid neighbors for each of the three template convolutions. These nearest neighbors falling within 3× *σ* of the template size were used to find the mean centroid position of individual Env clusters in the single assembly site. The final real-space centroid position was then determined from the maximum-likelihood that the Env cluster centroid contained the highest probability voxel value referenced relative to the highest probability voxel in the entire probability density. Env single-molecule localizations which were at least 2× *σ* of the maximum-likelihood centroid positions for each volume were stored. The final Env cluster-filtering routine passes the point cloud representation to a blinded supervising operator who then manually approves or disapproves individual Env clusters and centroids from three separately averaged convolutions for each virus assembly volume. Centroid positions were then used to calculate the angular (*φ*) distribution of individual Env clusters referenced from aligned membrane plane centroids and Gag cluster centroids.

### Total iPALM error assessment

The propagation of independent error arising from three-channel iPALM measurements and data processing are treated in Eq. 2 below:

2 $${{{\sigma_{{\rm{total}}}}} = \sqrt {\sigma^2_{\mathrm{drift}}+\sigma^2_{\mathrm{registration}} + \sigma^2_{\mathrm{AF750}} + \sigma^2_{\mathrm{AF647}} + \sigma^2_{\mathrm{PSCFP2}} + \sigma^2_{\mathrm{averaging,translational}}+\sigma^2_{\mathrm{averaging,rotational}}}}$$

The sample drift (*X*, *Y*, and *Z*) typically ranged from 5 to 50 nm for a given experiment. The residual *X*-, *Y*-, and *Z*-uncertainty after drift correction ranged from 1 to 5 nm (*σ*_drift_). Two-channel image registration typically resulted in a residual uncertainty of 3–8 nm (*σ*_registration_) in *X*-, *Y*-, and *Z*-dimensions depending on the density and quality of Au nanoparticles present in the field of view. Using the described iPALM method, localization uncertainties for all single-molecule detection events followed a lognormal distribution. The mean of the localization uncertainty distribution for the AlexaFluor 750 probe (*σ*_localization,AF750_) was typically around 10–15 nm in the *X*- and *Y*-dimensions and 5–12 nm in the *Z*-dimension. The mean of the localization uncertainty distribution for the AlexaFluor 647 probe (*σ*_localization,AF647_) was typically around 5–10 nm in the *X*- and *Y*-dimensions and 3–8 nm in the *Z*-dimension. Mean localization uncertainties for the PSCFP2 probe (*σ*_localization,PSCFP2_) ranged from 10 to 20 nm in *X*- and *Y*-dimensions and 10–15 nm in the *Z*-dimension. The residual translational uncertainty of alignment between the centroids of single HIV Gag clusters (*σ*_averaging,translational_) was no greater than 5-15 nm. The residual tilt uncertainty of alignment between the local plasma membrane planes (*σ*_averaging,translational_) was typically no greater than 3° as assessed by phase correlation lags of random half-sets of membrane plane probability densities (see Supplementary Fig. 11) or 4.39 ± 0.12° from Monte Carlo simulations (see Supplementary Fig. 10). Combined with the standard error estimated from weighted principle component analysis, we computed the Euclidean space error in rotationally aligned data sets to be about 10 nm in plane tilt uncertainty.

It is important to note that the real-space sampling density (Nyquist) for Env clusters is a convolution of the true Env density per particle (biologically sparse; 7–14 trimers per particle) and the number of particles averaged. Cross-correlation analysis of Env angular distributions (see Probability density analysis section above) suggested that sufficient sampling was present to distinguish surface density distributions in, at minimum, 20° intervals (largest cross-correlation lag in one data set). This is sufficient to conclude that Euclidean space shifts in Env angular probability densities between hemispheres are derived from biological changes in Env trimer distributions and therefore we do not consider the sampling resolution in this error assessment. Finally, the native Gag density per particle (3000–5000 molecules) yields sufficient sampling upon cluster averaging (typically up to 1–2 localizations per nm^3^; see ref.^22^) to render the sampling criteria negligible when compared with other errors in the system.

Collectively, under these data acquisition conditions, the localization uncertainties for the *X*- and *Y*-dimensions dominate the propagated errors. Specifically, those for the PSCFP2 fluorescent protein probe provides the largest uncertainty. The PSCFP2 probe was only used for calculation of the normal membrane plane vector, however, which is a parameter we found to be less sensitive to *X*- and *Y*-positional uncertainties because these errors were also affine transformed using the estimated normal vector for plane alignment. This Euclidean space error transformation served to lower the overall localization error by averaging σ_localization,*XY*_ with the much smaller interferometric *σ*_localization,__*Z*_. The total error measurement, *σ*_total_= 33.5 nm, is an estimate of the resolution of the system for the three-channel iPALM measurements.

### Single-molecule tracking analysis

Sparse labeling of HIV-1 Env on the surface of cellular specimens presented as well-resolved diffraction-limited spots, which displayed the expected single-step photobleaching characteristics of quantized fluorophore bleaching (average degree of labeling was 1 Atto565 dye per Fab b12). Detection of single-molecule centroids and residual uncertainty was computed identically to the protocol described in the iPALM method. Localization position estimates over the frame interval were then linked using modified scripts from the tracking algorithms found in the Matlab package uTrack^24^. The mean localization uncertainty for these experimental conditions was 14 nm, but the uncertainty of each localization was utilized on a per-track basis for linking localizations. Only tracks with greater than 20 positions were used to compute the the short-range diffusion coefficient for each track (*D* = μm^2^ s^−1^). Tracks were classified as mobile or confined/immobile based on the uTrack classification system. Briefly, uTrack classifications were assigned using moment orders of 0 through 6, and a MSS analysis algorithm as described previously^24,42^. Tracks were conservatively grouped as immobile if uTrack classification assigned either a 0 (immobile) or 1 (confined Brownian) diffusion classification. Only tracks classified as demonstrating “pure Brownian” motion were grouped as mobile for the purposes of our analysis.

### Code availability

Custom MATLAB code is available from the authors on request, in total or in modules.

### Data availability

All data supporting the findings of this study are available within the article and its Supplementary Information files, or are available from the authors on request.

## Electronic supplementary material

Suppplementary Information

## References

[1] Freed EO (2015). HIV-1 assembly, release and maturation. Nat. Rev. Microbiol..

[2] Sundquist WI, Krausslich HG (2012). HIV-1 assembly, budding, and maturation. Cold Spring Harb. Perspect. Med..

[3] Hanne J, Zila V, Heilemann M, Muller B, Krausslich HG (2016). Super-resolved insights into human immunodeficiency virus biology. FEBS Lett..

[4] Muranyi W, Malkusch S, Muller B, Heilemann M, Krausslich HG (2013). Super-resolution microscopy reveals specific recruitment of HIV-1 envelope proteins to viral assembly sites dependent on the envelope C-terminal tail. PLoS Pathog..

[5] Murakami T, Freed EO (2000). The long cytoplasmic tail of gp41 is required in a cell type-dependent manner for HIV-1 envelope glycoprotein incorporation into virions. Proc. Natl Acad. Sci. USA.

[6] Akari H, Fukumori T, Adachi A (2000). Cell-dependent requirement of human immunodeficiency virus type 1gp41 cytoplasmic tail for Env incorporation into virions. J. Virol..

[7] Freed EO, Martin MA (1995). Virion incorporation of envelope glycoproteins with long but not short cytoplasmic tails is blocked by specific, single amino acid substitutions in the human immunodeficiency virus type 1 matrix. J. Virol..

[8] Mammano F, Kondo E, Sodroski J, Bukovsky A, Gottlinger HG (1995). Rescue of human immunodeficiency virus type 1 matrix protein mutants by envelope glycoproteins with short cytoplasmic domains. J. Virol..

[9] Zhu P (2003). Electron tomography analysis of envelope glycoprotein trimers on HIV and simian immunodeficiency virus virions. Proc. Natl Acad. Sci. USA.

[10] Tedbury PR, Novikova M, Ablan SD, Freed EO (2016). Biochemical evidence of a role for matrix trimerization in HIV-1 envelope glycoprotein incorporation. Proc. Natl Acad. Sci. USA.

[11] Chojnacki J (2017). Envelope glycoprotein mobility on HIV-1 particles depends on the virus maturation state. Nat. Commun..

[12] Roy NH, Chan J, Lambele M, Thali M (2013). Clustering and mobility of HIV-1 Env at viral assembly sites predict its propensity to induce cell-cell fusion. J. Virol..

[13] Alfadhli A, Barklis RL, Barklis E (2009). HIV-1 matrix organizes as a hexamer of trimers on membranes containing phosphatidylinositol-(4,5)-bisphosphate. Virology.

[14] Murakami T, Freed EO (2000). Genetic evidence for an interaction between human immunodeficiency virus type 1 matrix and alpha-helix 2 of the gp41 cytoplasmic tail. J. Virol..

[15] Tedbury PR, Ablan SD, Freed EO (2013). Global rescue of defects in HIV-1 envelope glycoprotein incorporation: implications for matrix structure. PLoS Pathog..

[16] Qi M (2013). Rab11-FIP1C and Rab14 direct plasma membrane sorting and particle incorporation of the HIV-1 envelope glycoprotein complex. PLoS Pathog..

[17] Tremblay M (1989). New CD4(+) cell line susceptible to infection by HIV-1. J. Med. Virol..

[18] Huang M, Orenstein JM, Martin MA, Freed EO (1995). p6Gag is required for particle production from full-length human immunodeficiency virus type 1 molecular clones expressing protease. J. Virol..

[19] Freed EO (2002). Viral late domains. J. Virol..

[20] Gottlinger HG, Dorfman T, Sodroski JG, Haseltine WA (1991). Effect of mutations affecting the p6 gag protein on human immunodeficiency virus particle release. Proc. Natl Acad. Sci. USA.

[21] Shtengel G (2009). Interferometric fluorescent super-resolution microscopy resolves 3D cellular ultrastructure. Proc. Natl Acad. Sci. USA.

[22] Van Engelenburg SB (2014). Distribution of ESCRT machinery at HIV assembly sites reveals virus scaffolding of ESCRT subunits. Science.

[23] Sochacki KA, Shtengel G, van Engelenburg SB, Hess HF, Taraska JW (2014). Correlative super-resolution fluorescence and metal-replica transmission electron microscopy. Nat. Methods.

[24] Jaqaman K (2008). Robust single-particle tracking in live-cell time-lapse sequences. Nat. Methods.

[25] Egan MA, Carruth LM, Rowell JF, Yu X, Siliciano RF (1996). Human immunodeficiency virus type 1 envelope protein endocytosis mediated by a highly conserved intrinsic internalization signal in the cytoplasmic domain of gp41 is suppressed in the presence of the Pr55gag precursor protein. J. Virol..

[26] Chojnacki J (2012). Maturation-dependent HIV-1 surface protein redistribution revealed by fluorescence nanoscopy. Science.

[27] Qi M (2015). A tyrosine-based motif in the HIV-1 envelope glycoprotein tail mediates cell-type- and Rab11-FIP1C-dependent incorporation into virions. Proc. Natl Acad. Sci. USA.

[28] Stano, A. et al. Dense array of spikes on HIV-1 virion particles. *J. Virol*. **91**, e00415-17 (2017). 10.1128/JVI.00415-17 PMC5487557 28446665

[29] Rusert P (2016). Determinants of HIV-1 broadly neutralizing antibody induction. Nat. Med..

[30] Barbas CF (1992). Recombinant human Fab fragments neutralize human type 1 immunodeficiency virus in vitro. Proc. Natl Acad. Sci. USA.

[31] Burton DR (1994). Efficient neutralization of primary isolates of HIV-1 by a recombinant human monoclonal antibody. Science.

[32] Burton DR (1991). A large array of human monoclonal antibodies to type 1 human immunodeficiency virus from combinatorial libraries of asymptomatic seropositive individuals. Proc. Natl Acad. Sci. USA.

[33] Roben P (1994). Recognition properties of a panel of human recombinant Fab fragments to the CD4 binding site of gp120 that show differing abilities to neutralize human immunodeficiency virus type 1. J. Virol..

[34] Trkola A (1996). Human monoclonal antibody 2G12 defines a distinctive neutralization epitope on the gp120 glycoprotein of human immunodeficiency virus type 1. J. Virol..

[35] Buchacher A (1994). Generation of human monoclonal antibodies against HIV-1 proteins; electrofusion and Epstein-Barr virus transformation for peripheral blood lymphocyte immortalization. AIDS Res. Hum. Retrovir..

[36] Barbas CF (1993). Molecular profile of an antibody response to HIV-1 as probed by combinatorial libraries. J. Mol. Biol..

[37] Huang J (2012). Broad and potent neutralization of HIV-1 by a gp41-specific human antibody. Nature.

[38] Gao F (2009). Cross-reactive monoclonal antibodies to multiple HIV-1 subtype and SIVcpz envelope glycoproteins. Virology.

[39] Brown TA (2011). Superresolution fluorescence imaging of mitochondrial nucleoids reveals their spatial range, limits, and membrane interaction. Mol. Cell. Biol..

[40] Betzig E (2006). Imaging intracellular fluorescent proteins at nanometer resolution. Science.

[41] Heilemann M (2008). Subdiffraction-resolution fluorescence imaging with conventional fluorescent probes. Angew. Chem..

[42] Ewers H (2005). Single-particle tracking of murine polyoma virus-like particles on live cells and artificial membranes. Proc. Natl Acad. Sci. USA.

