# Supplementary material for: Single molecule fate of HIV-1 envelope reveals late-stage viral lattice incorporation
Source: Nat Commun. 2018 May 10;9:1861. doi: 10.1038/s41467-018-04220-w (PMC5945595; doi:10.1038/s41467-018-04220-w)
Supplement: Supplementary file 1 — Suppplementary Information [file 41467_2018_4220_MOESM1_ESM.pdf]

Supplementary Information for:

"Single molecule fate of HIV-1 envelope reveals late-stage viral lattice incorporation"

Carmen A. Buttler[1,4], Nairi Pezeshkian[1,4], Melissa V. Fernandez[2], Jesse Aaron[3], Sofya Norman[1], Eric O. Freed[2], and Schuyler B. van Engelenburg[1*]

* Corresponding author (schuyler.vanengelenburg@du.edu).

1. Molecular and Cellular Biophysics Program, Department of Biological Sciences, University of Denver, Denver, CO, USA, 80210.
2. HIV Dynamics and Replication Program, Center for Cancer Research, National Cancer Institute, Frederick, MD, USA, 21702.
3. Advanced Imaging Center, Howard Hughes Medical Institute, Janelia Research Campus, Ashburn, VA, USA, 20147.
4. These authors contributed equally to this work

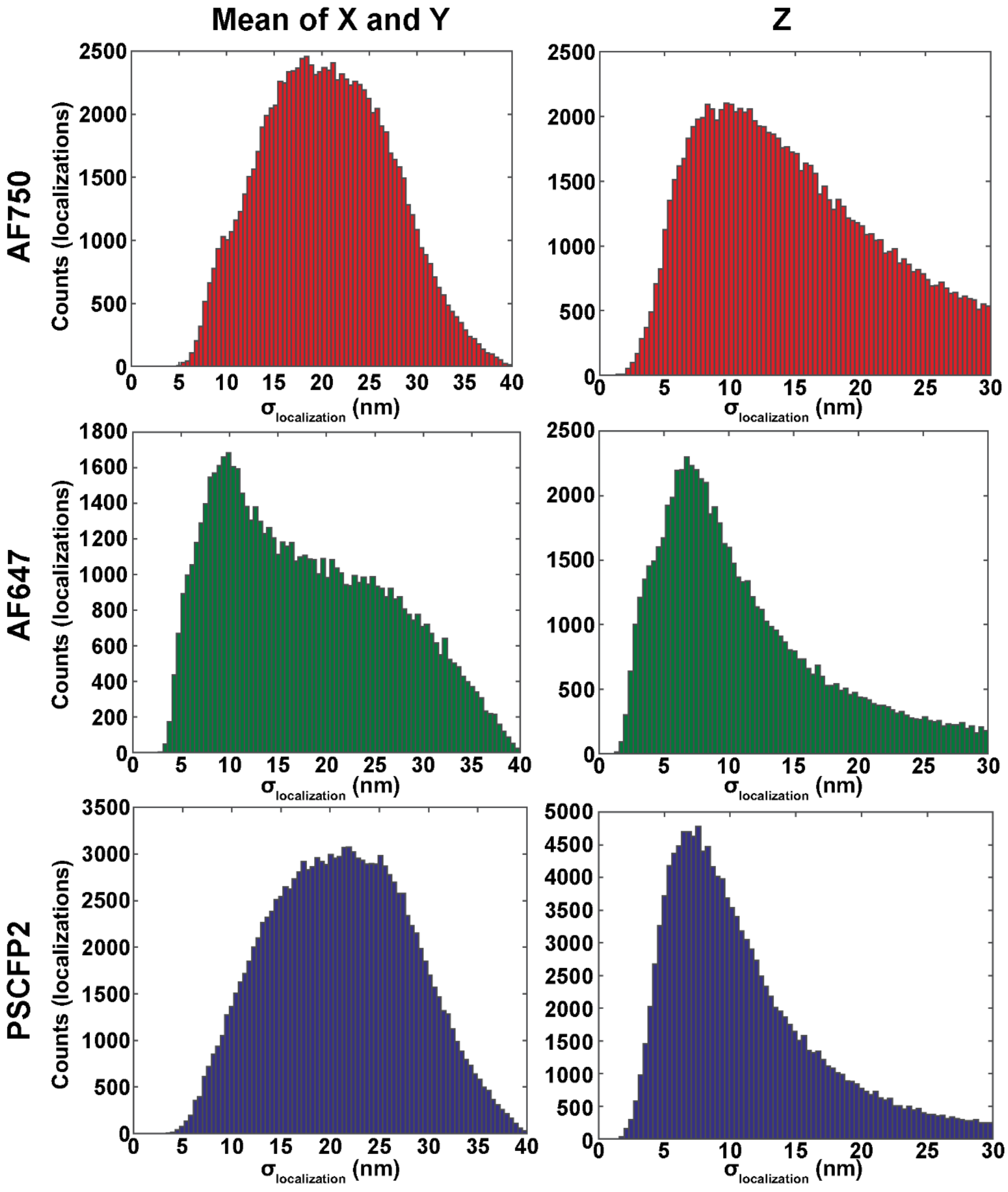

Supplementary Fig. 1. Representative σ values of localization precision for the fluorophores used with iPALM measurements. Representative histograms of the sigma values for the X-, Y- (mean) and Z-dimensions associated with each single molecule localization from all buds segmented from COS7 cells expressing d8-Env (the largest dataset). Each channel in all datasets was filtered based on $\sigma_{\text{localization, x,y,z}}$ to remove any localizations with greater than 40 nm uncertainty in the X-, Y-dimensions and 30 nm uncertainty for the Z-dimension. For AlexaFluor750 (AF750, top) mean X,Y = 20.6±6.6 nm (left) and Z = 14.6±6.6 nm (right). For AlexaFluor647 (AF647, middle) mean X,Y = 18.0±8.7 nm (left) and Z = 11.0±6.3 nm (right). For PSCFP2 (bottom) mean X,Y = 21.1±7.0 nm (left), and Z = 11.0±5.7 nm (right). Error of the mean represents s.d.

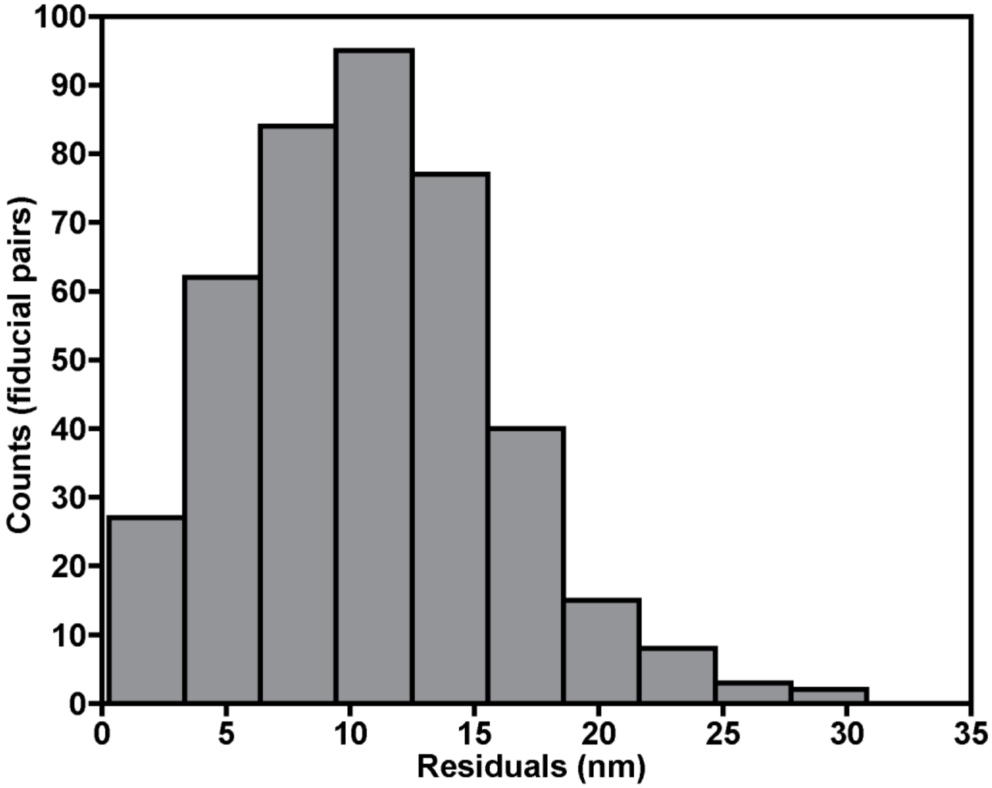

Supplementary Fig. 2. Randomly sampled residual uncertainty for fiducial pairs after linear affine transform of one superresolution imaging channel onto another. Au nanorods, constitutively fluorescent in all three channels, were used as fiducial points for registration of the three channels. The residual error in fiducial centroids s.d. = $\sigma_{registration}$ = 5.2 nm. n = 413 fiducials pairs over 19 imaging experiments. The mean registration uncertainty was used as a seed parameter in simulations for estimating the total error in angular measurements (φ) of Env.

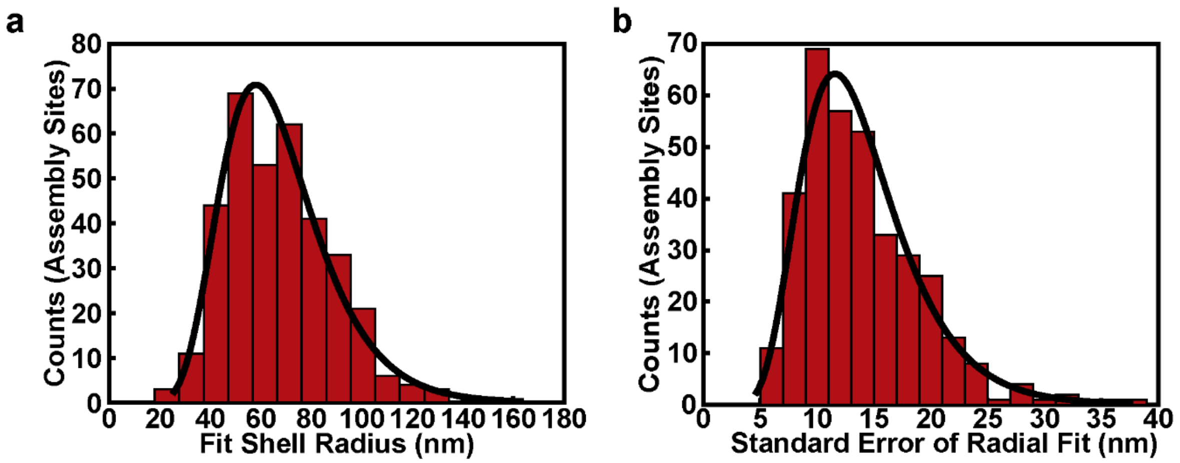

Supplementary Fig. 3. Estimated Gag lattice radius and residual error for a representative dataset. **(a)** Distribution of the fit radius of a random set of 350 Gag clusters. 70±22 nm. **(b)** Distribution of the fit uncertainty of the same random set of 350 representative Gag clusters, means = 13.9±5.1 nm. Error is represented as s.d. This residual error in Gag lattice centroid position was used as an estimate of the uncertainty in translational particle alignment and as a seed parameter in the described simulations.

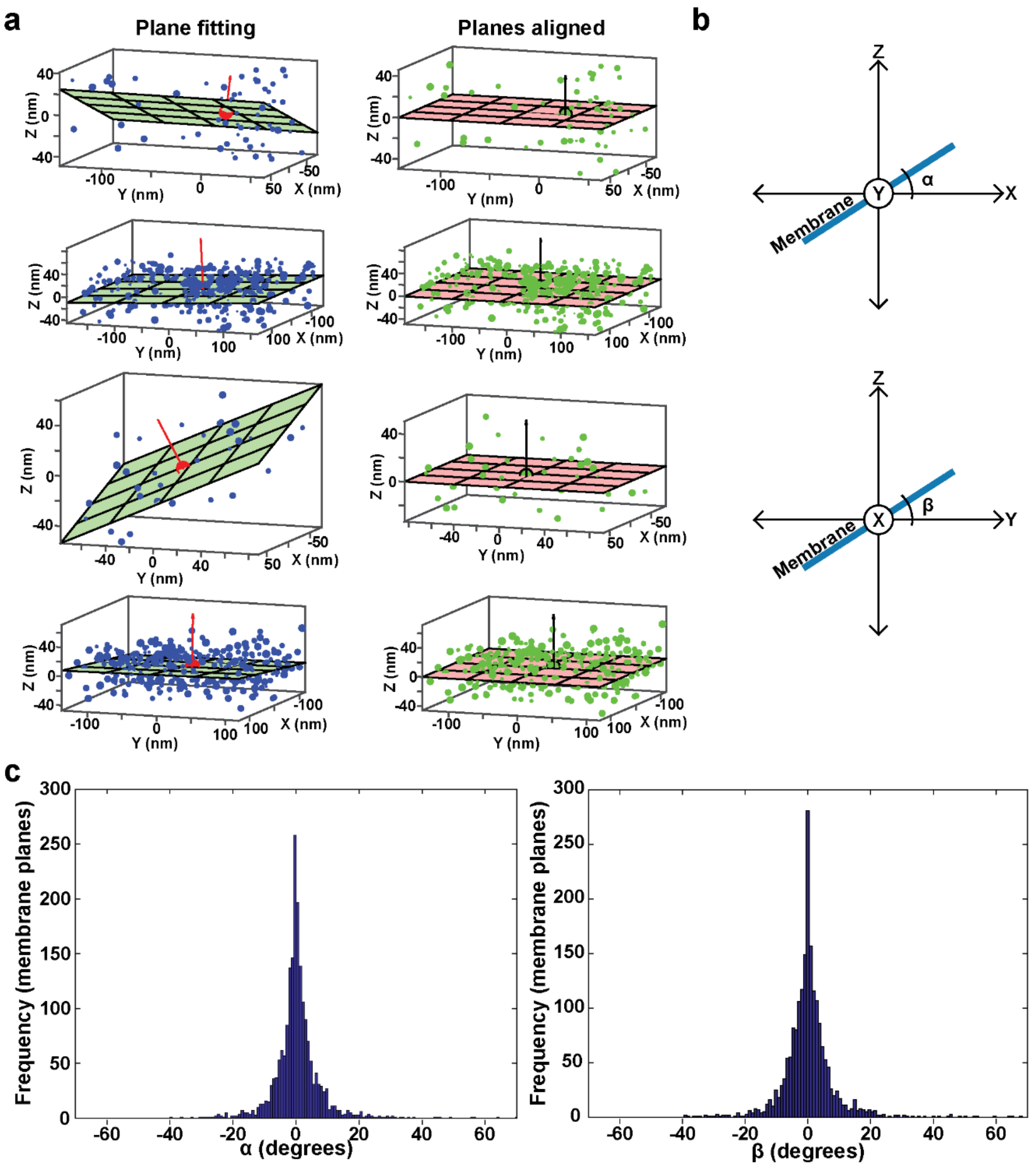

Supplementary Fig. 4. Weighted principle component analysis (PCA) was used to fit local membrane planes at single HIV-1 assembly sites. (a) Representative examples of local plasma membrane signals arising from S15-PSCFP2 labeling at segmented individual virus assembly sites (200×200 - 400×400 nm). These local membrane planes described the budding polarity of each virus assembly site and were used to rotationally align all segmented particles. Plane fits before (left; green) and after orthogonal alignment with the optical axis Z (right; pink). The normal vectors describing the budding profiles before (left; red vector) and after alignment (right; black vector). The localization precisions for each centroid were used as weights for PCA. **(b)** Definition of the angles α and β as the X- and Y-components of tilt, respectively. **(c)** The α and β angles estimated from weighted PCA for all segmented HIV-1 assembly sites analyzed in this study. The distributions demonstrate that the nanoscale budding polarity for assembly sites is diverse and requires alignment prior to virus cluster averaging.

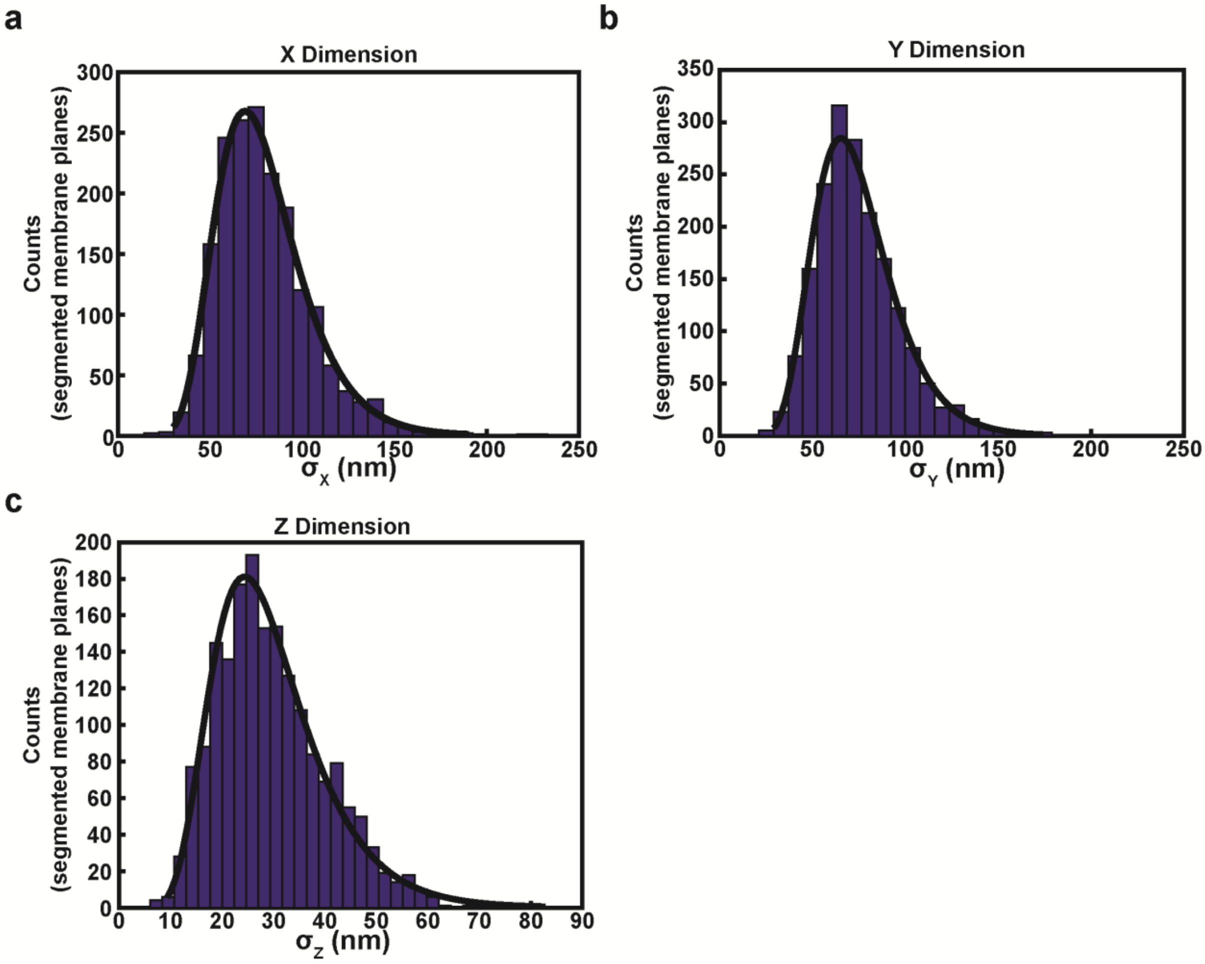

Supplementary Fig. 5. Distributions of segmented plasma membrane sizes after plane normalization to the microscopic axis were used to generate simulation seed parameters for error estimation. (a) Distribution of standard deviations of localization distances in the X dimension (σ_x; mean = 79.3 ± 25.0 nm). **(b)** Distribution of standard deviations of localization distances in the Y dimension (σ_y; mean = 74.5 ± 22.7 nm). **(c)** Distribution of standard deviations of distances in the Z dimension normal to the fitted plane (σ_z; mean =29.7 ± 11.2 nm). Axis dimensions are relative to the microscope axis. Error is reported as s.d. of log normal fits. n = 1837 plasma membrane planes labelled with S15-PSCFP2.

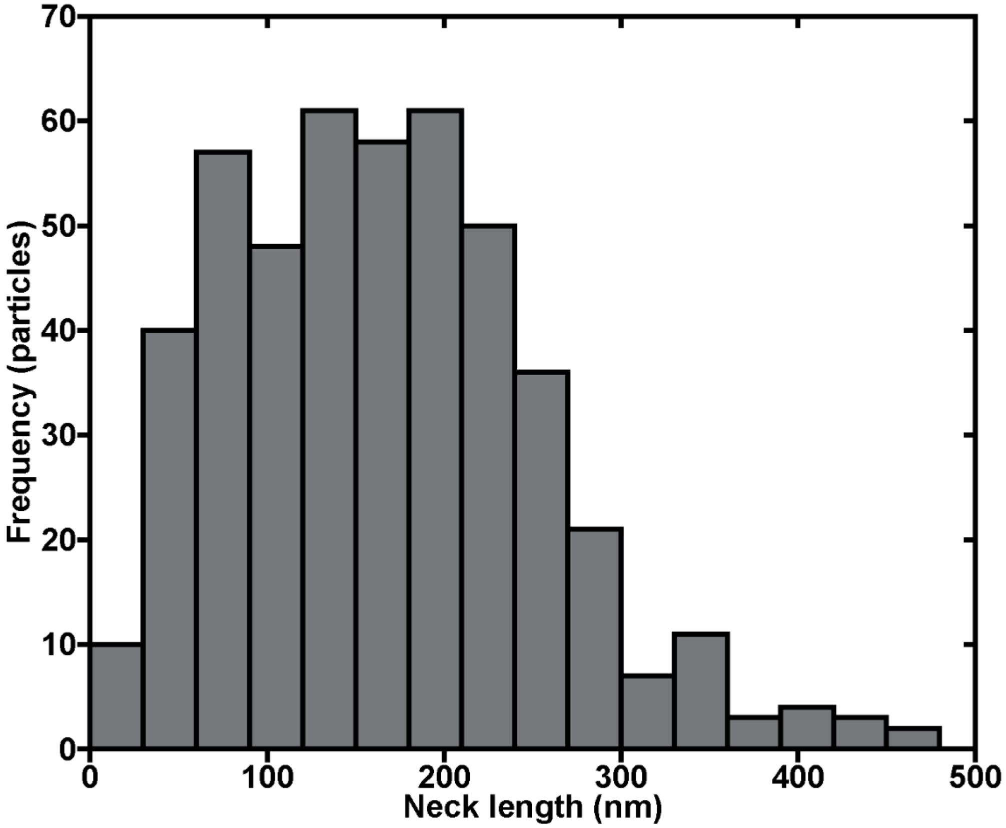

Supplementary Fig. 6. Distribution of the virus bud neck lengths demonstrates expected distances for cell associated particles. Representative histogram of the neck lengths of budding particles from a single dataset. The virus bud neck length was estimated by calculation of the distance between the centroid of the Gag shell fit center and the centroid of the local membrane plane after plane alignment with the optical axis (Z). For this dataset, the mean virus bud neck length was 165±86 nm (n = 474 assembly sites). For all segmented data, the mean was 150±92 nm (n = 1837 assembly sites) which is in good agreement with the bud neck lengths estimated for release-defective (*ΔPTAP*) HIV-1 by transmission electron microscopy (127±38 nm, see Fig. S15). Error represents s.d. from the mean.

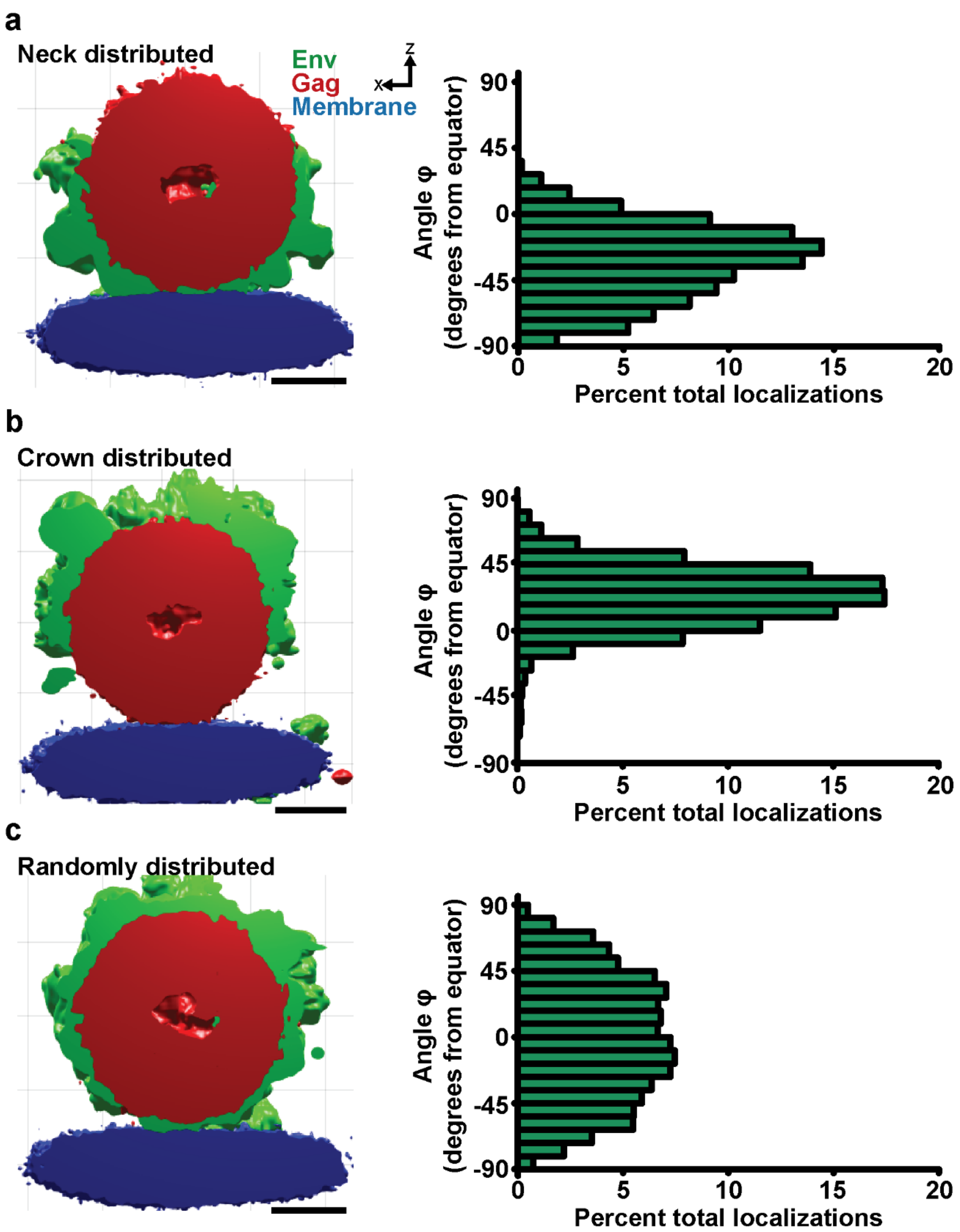

Supplementary Fig. 7. Simulated HIV-1 Env clusters are quantitatively reconstructed by the alignment and angular distribution software pipeline. Simulation of angularly-biased Env cluster datasets, using seed parameters which closely mimic biological datasets, are faithfully reconstructed. The imposed angular bias for each dataset is evident from integrated angular probability densities. Synthetic virus assembly sites were generated with **(a)** a neck-distribution (φ center at -45°), **(b)** a crown-distribution (φ centered at 45°), or **(c)** a random-distribution (normally distributed φ). Scale bars represent 100 nm, n = 200 for each dataset, and bin widths are 9°.

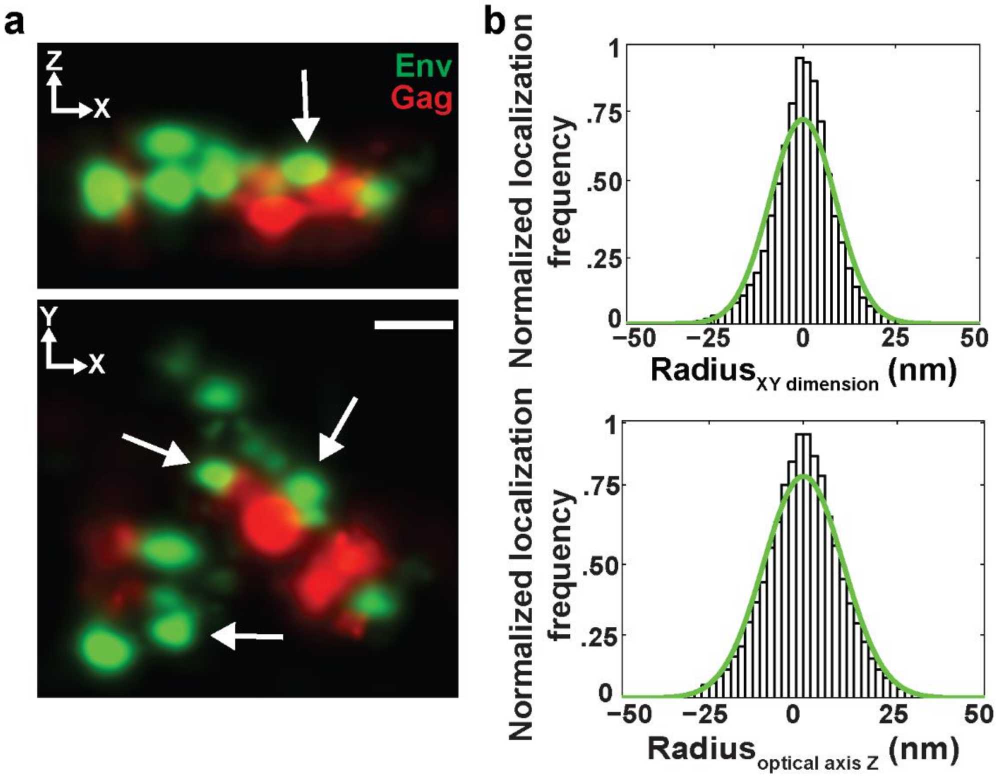

Supplementary Fig. 8. Geometric estimates of Env clusters were manually assessed to guide creation of simulation and segmentation algorithms. (a) iPALM data from a COS7 cell expressing WT-Env at sites of three Gag clusters (red) showing peripheral localization of Env clusters (green). The iPALM micrographs depict resolution of individual Env clusters (white arrows) along the X-, Y-, and Z-dimensions. Scale bar is 100 nm. **(b)** Probability distributions of HIV-1 Env clusters analyzed for 400 Gag-positive assembly sites. The standard deviations ($\sigma_{\text{geometric clusters}}$) for the X- and Z-dimensions were calculated to be 9.3 nm and 11.0 nm from a Gaussian fit of the positional centroids of all clusters, respectively (Y-dimension was the same as the X-dimension and is not shown). The cluster dimensions are approaching the resolution limit of the iPALM method (see Total Error Assessment section of the methods). Given the low density of Env clusters observed per particle (see Fig. S9) and an average of 7-14 trimers per HIV-1 particle[1] this suggests that trimers physically cluster on the surface of budding particles. These clusters, nevertheless, are readily resolvable at single assembly sites if separated by at least $2\times\ \sigma_{\text{geometric clusters}}$.

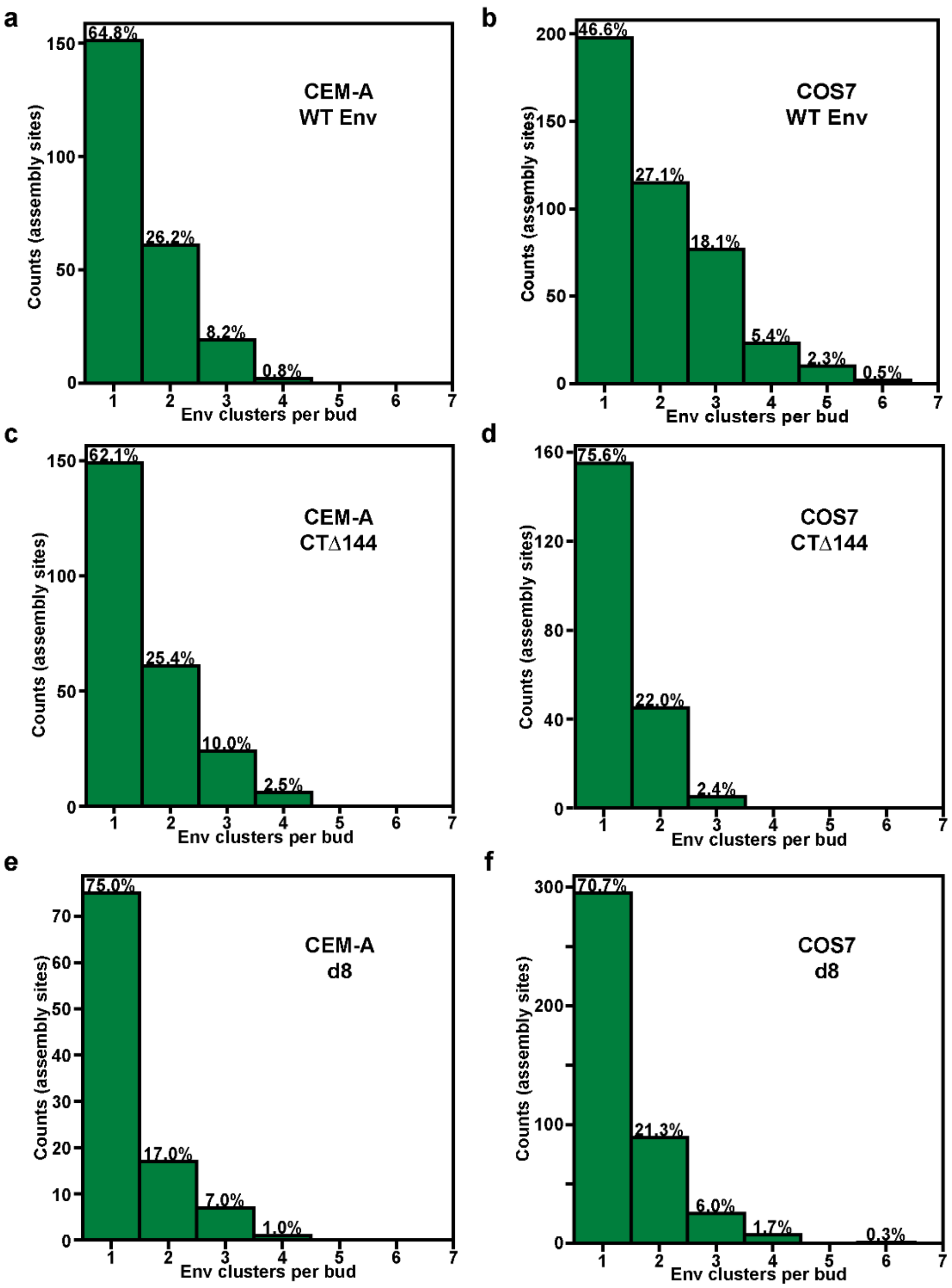

Supplementary Fig. 9. Numbers of discretely measured Env clusters per particle supports model of Env clusters as accumulations of multiple trimers. Observed distributions of Env clusters per particle agree well with previous imaging studies[2,3] but do not account for the 7-14 trimers per particle observed by electron microscopy [1], which supports the suggestion that clusters represent accumulations of multiple trimers. **(a)** Histogram of particles segmented from CEM-A cells producing WT-Env with 1, 2, 3, or 4 discretely measured Env clusters **(b)** Histogram of particles segmented from COS7 cells producing WT-Env with 1, 2, 3, 4, 5, or 6 Env clusters **(c)** Histogram of particles segmented from CEM-A cells producing CTΔ144-Env with 1, 2, 3, or 4 Env clusters. **(d)** Histogram of particles segmented from COS7 cells producing CTΔ144-Env with 1, 2, or 3 Env clusters. **(e)** Histogram of particles segmented from CEM-A cells producing d8-Env with 1, 2, 3, or 4 Env clusters. **(f)** Histogram of particles segmented from COS7 cells producing d8-Env with 1, 2, 3, 4, 5, or 6 Env clusters.

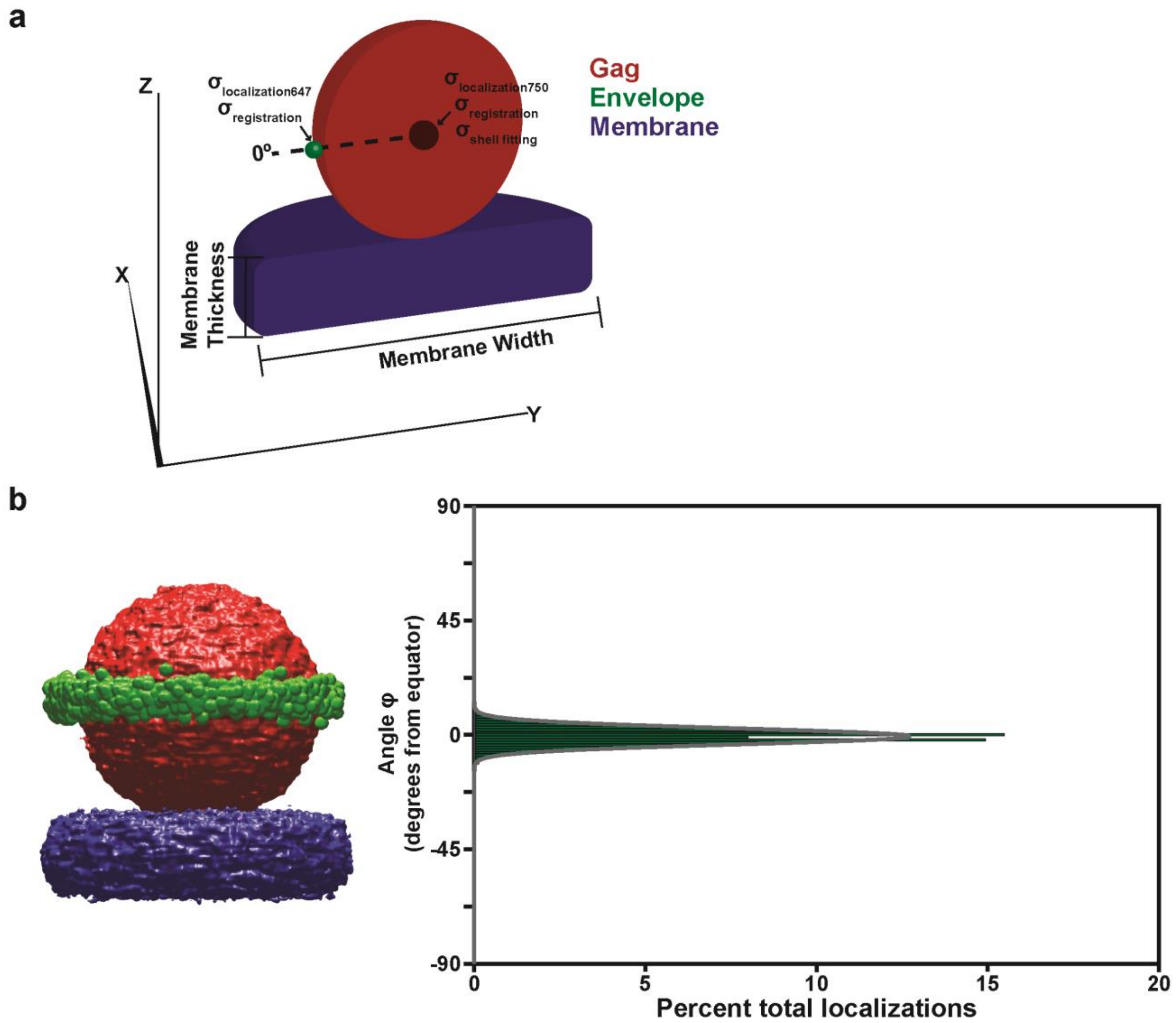

Supplementary Fig. 10. Monte Carlo simulation of the total error in estimation of Env angular distribution is no greater than 5°. (a) A cartoon depicting the sources of error in registration, shell fitting, localization, and membrane plane estimation. Gag clusters were generated with a random radius sampled from the distribution derived from the fit in Fig. S3 (red sphere). Membrane planes were randomly simulated using seed parameters and variance measured using empirical data (Fig. S5a-b; blue disc). Membrane planes were randomly offset from the centroid of Gag clusters using a distribution measured empirically from the estimated neck length (Fig. S6). Each Gag cluster was then generated with a single Env localization at a fixed equatorial angle of $\varphi = 0°$. All three localization channels were then rotated randomly about the centroid of Gag (plane angles α and β; Fig. S4b-c). Random localization precisions were assigned from the distributions measured for the three chromophore labels. The resulting simulated virus assembly sites (n = 2500) were then processed through the same data processing pipeline as our experimental data. (**b**) The resulting probability density from single assembly site averaging is depicted as an isosurfaced object (left) for Gag (red), Env (green), and plasma membrane (blue). Calculation of the percent of total simulated Env localizations relative to the elevation angle φ resulted in a standard deviation of $4.39 \pm 0.12°$ from the fixed simulation value of 0°, but due to the compounding errors of localization, channel alignment, centroid fitting, and plane fitting. Isosurfaced thresholds are 1×10^{-1} (Gag), 1.0×10^{-4} (Env), 1.5×10^{-1} (plasma membrane) localizations × nm^3.

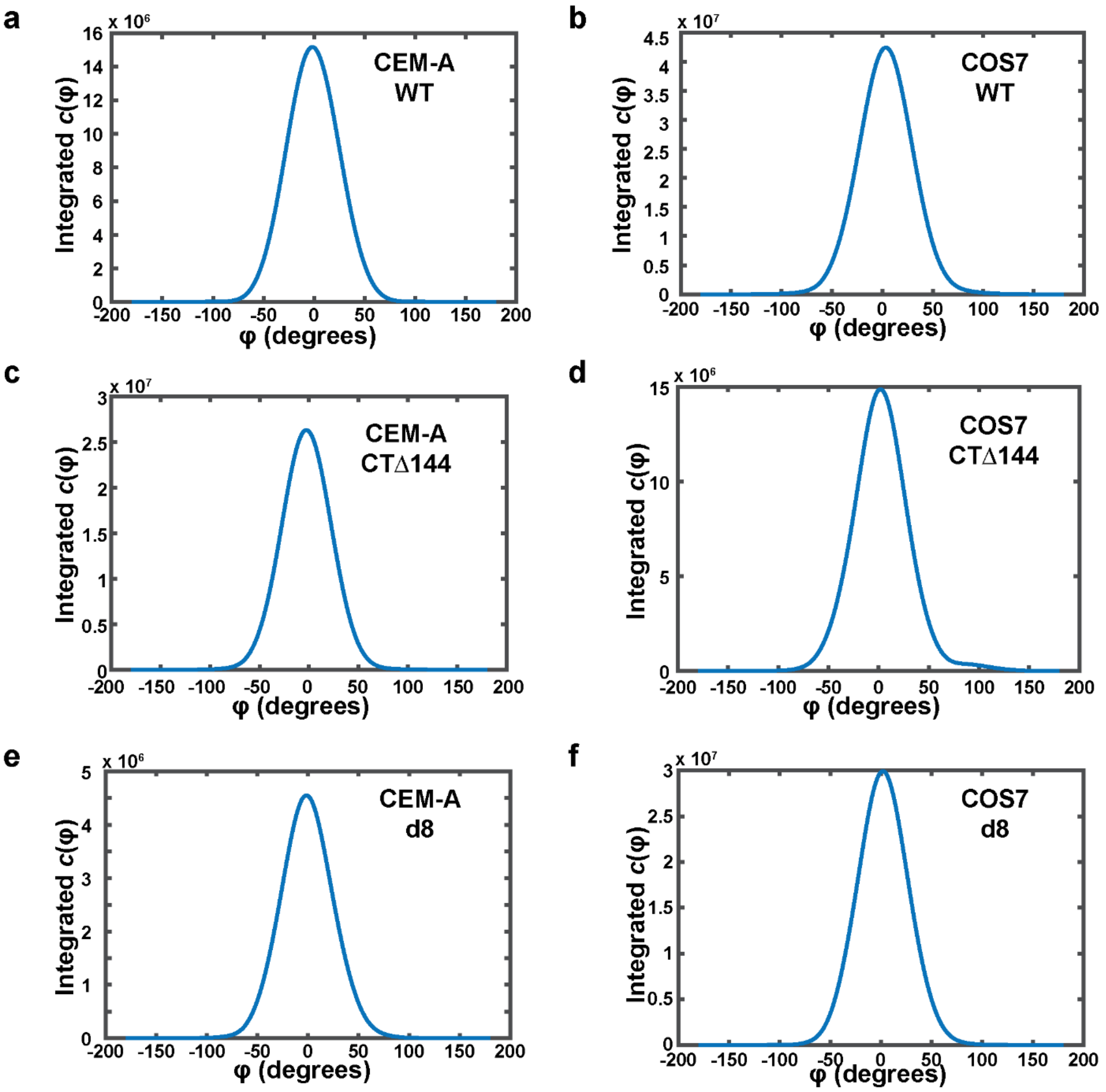

Supplementary Fig. 11. Cross-correlation of the S15-PSCFP2 membrane signal versus the elevation angle of the plane (φ) estimates the error in tilt alignment of all rotationally aligned membrane planes. Cross-correlation between the membrane planes of randomly divided half-datasets produces a small, but detectable lag between peak correlation and perfectly aligned radial probability density vectors (φ = 0°). This lag is an estimate of the tilt alignment and sampling of the probability densities describing the local membrane plane around averaged assembly sites. **(a)** CEM-A cells expressing WT-Env, the lag from maximum correlation and perfect alignment is 2°, n = 265 planes. **(b)** For COS7 cells expressing WT-Env, lag = 3°, n = 454 planes. **(c)** For CEM-A cells expressing CTΔ144-Env, lag = 3°, n = 276 planes. **(d)** For COS7 cells expressing CTΔ144-Env, lag = 2°, n = 244 planes. **(e)** For CEM-A cells expressing d8-Env, lag = 1°, n = 124 planes. **(f)** For COS7 cells expressing d8-Env, lag = 2°, n = 474 planes.

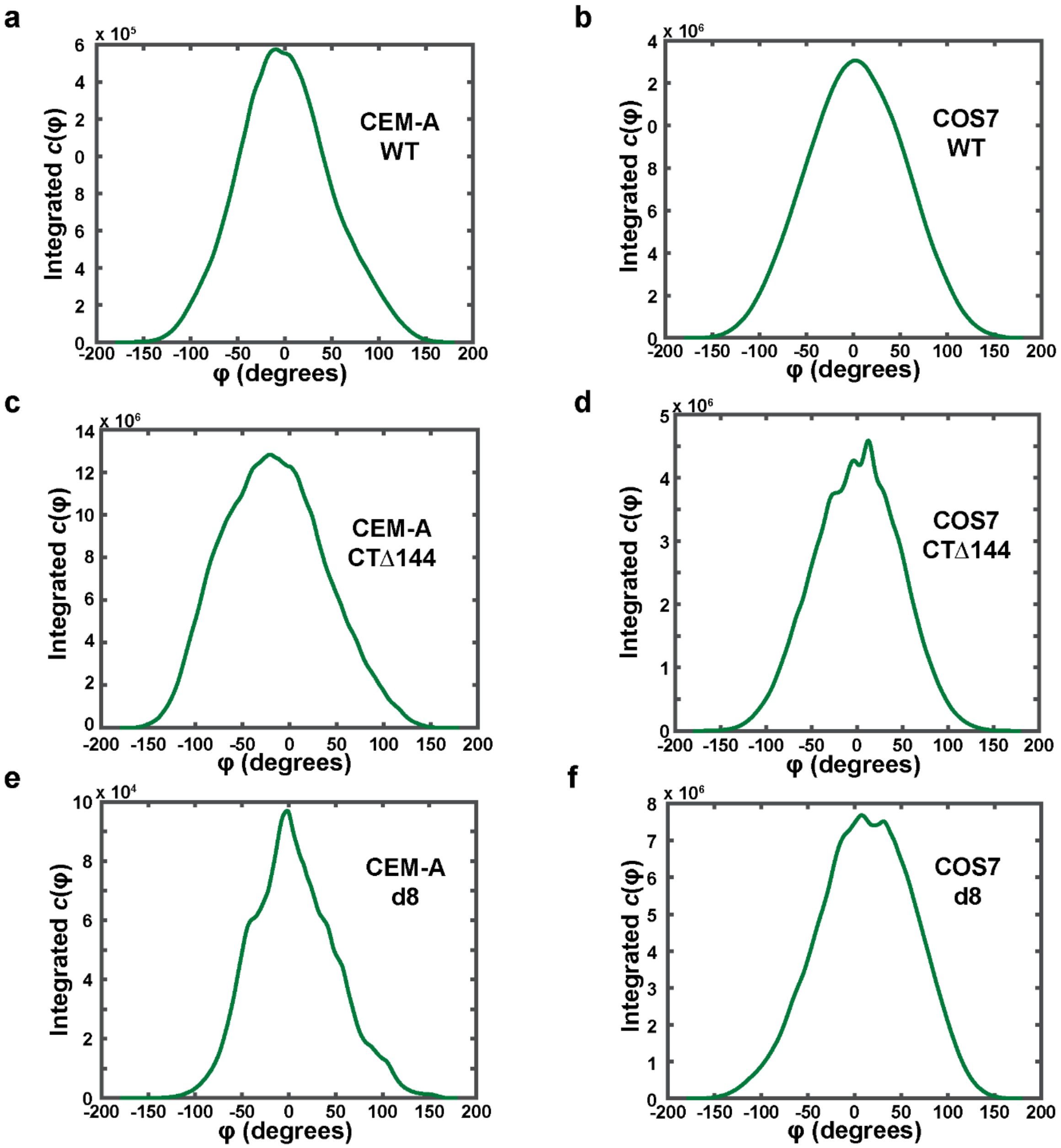

Supplementary Fig. 12. Cross-correlation of the angular (φ) densities of Env between random half-datasets determines the sampling resolution of the Env channel. The ability to distinguish changes in angular probability density over the surface of a sphere is dictated by the sampling resolution or signal frequency over that sphere surface. Cross-correlation of the integrated angular probability distribution of Env signal using random half-datasets provides an estimate of the sampling resolution of the system. **(a)** CEM-A cells producing WT-Env, the lag from maximum correlation and perfect alignment (0°) = -20° (n = 265 particles). **(b)** COS7 cells producing WT-Env, lag = 2° (n = 454 particles). **(c)** CEM-A cells producing CTΔ144-Env, lag = -20° (n = 276 particles). **(d)** COS7 cells producing CTΔ144-Env, lag = 8° (n = 244 particles). **(e)** CEM-A cells producing d8-Env, lag = -2° (n = 124 particles). **(f)** COS7 cells producing d8-Env, lag = 8° (n = 474 particles). The mean of the absolute value for all the lags over all datasets was φ = 9°, which was used as the histogram bin resolution for the angular probability vectors of φ over all datasets (see Fig. 2b and Fig. S13, S14).

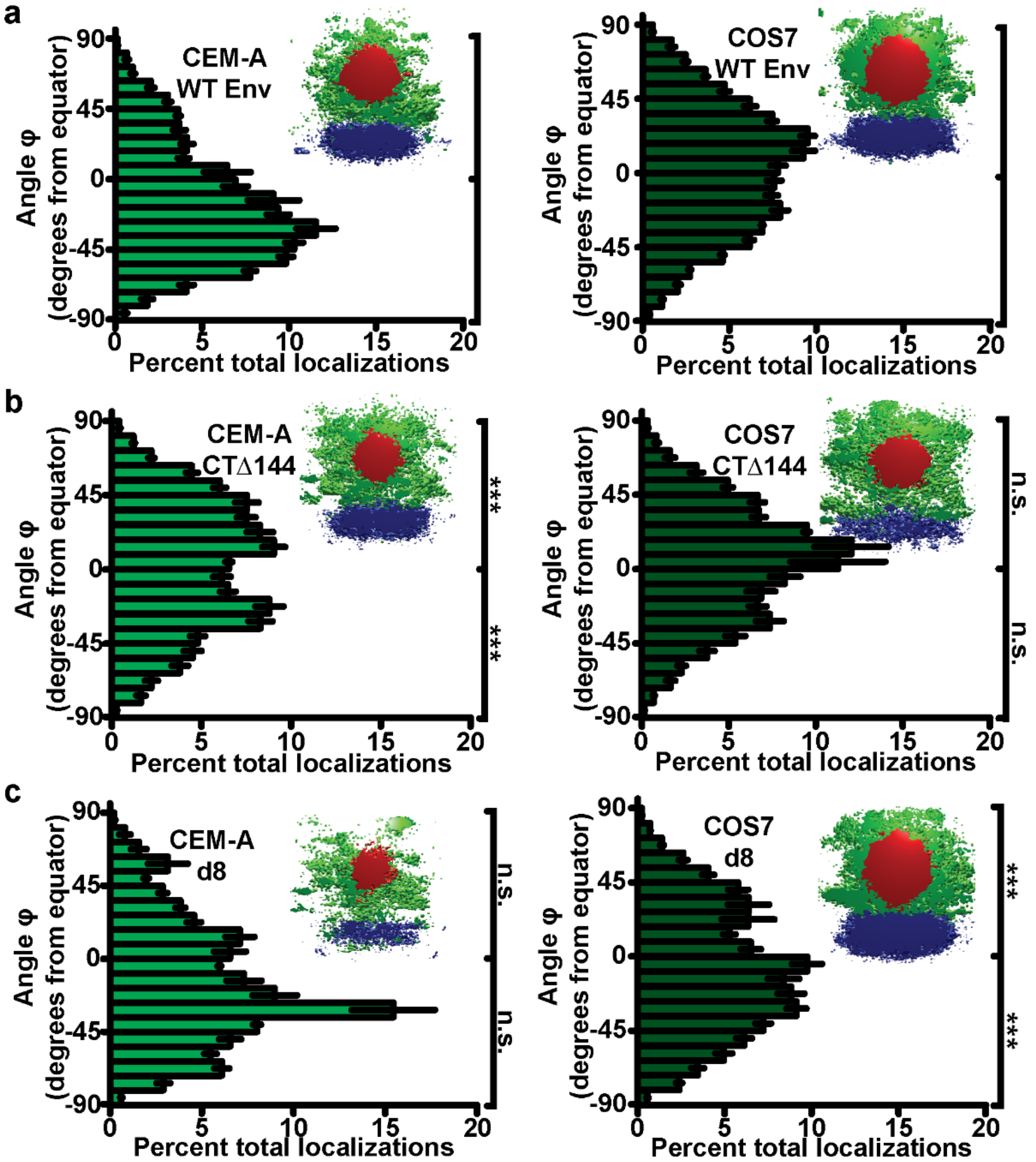

Supplementary Fig. 13. Angular probability densities for distributions of Env in each cell type and genotype of the Env-CT. Probability densities of aligned buds from each condition were integrated along the cone representing each elevation angle φ. Values of φ were binned in 9° increments according to the mean estimated sampling resolution of all datasets (see Fig. S12). Insets are cross-sections of each probability density for the three aligned channels. Bar graphs represent total probability density of φ, at a bandwidth of 9°, integrated along the radial and θ vectors. The probability density was normalized as a percentage of the total probability for all virus assembly sites in that condition. Error bars represent s.d. of three pairs of randomly divided half-datasets. **(a)** The φ distribution of WT-Env is biased toward the neck of virus buds in CEM-A (left, n = 265 particles from 3 cells) and unbiased when produced in COS7 cells (right, n = 454 particles from 5 cells). **(b)** Distribution of CTΔ144-Env is unbiased when produced in both CEM-A (left, n = 276 particles from 4 cells) and COS7 cells (right, n = 244 particles from 3 cells). **(c)** Distribution of d8-Env is neck-biased when produced by both CEM-A (left, n = 124 particles from 4 cells) and COS7 cells (right, n = 474 particles from 4 cells). Isosurface thresholds were 3.0×10^{-3} (Gag; red), 1.0×10^{-3} (Env; green), 4.5×10^{-3} (membrane; blue) localizations × nm^3. *** = $P < 0.001$ using two-way ANOVA and Bonferroni post-test.

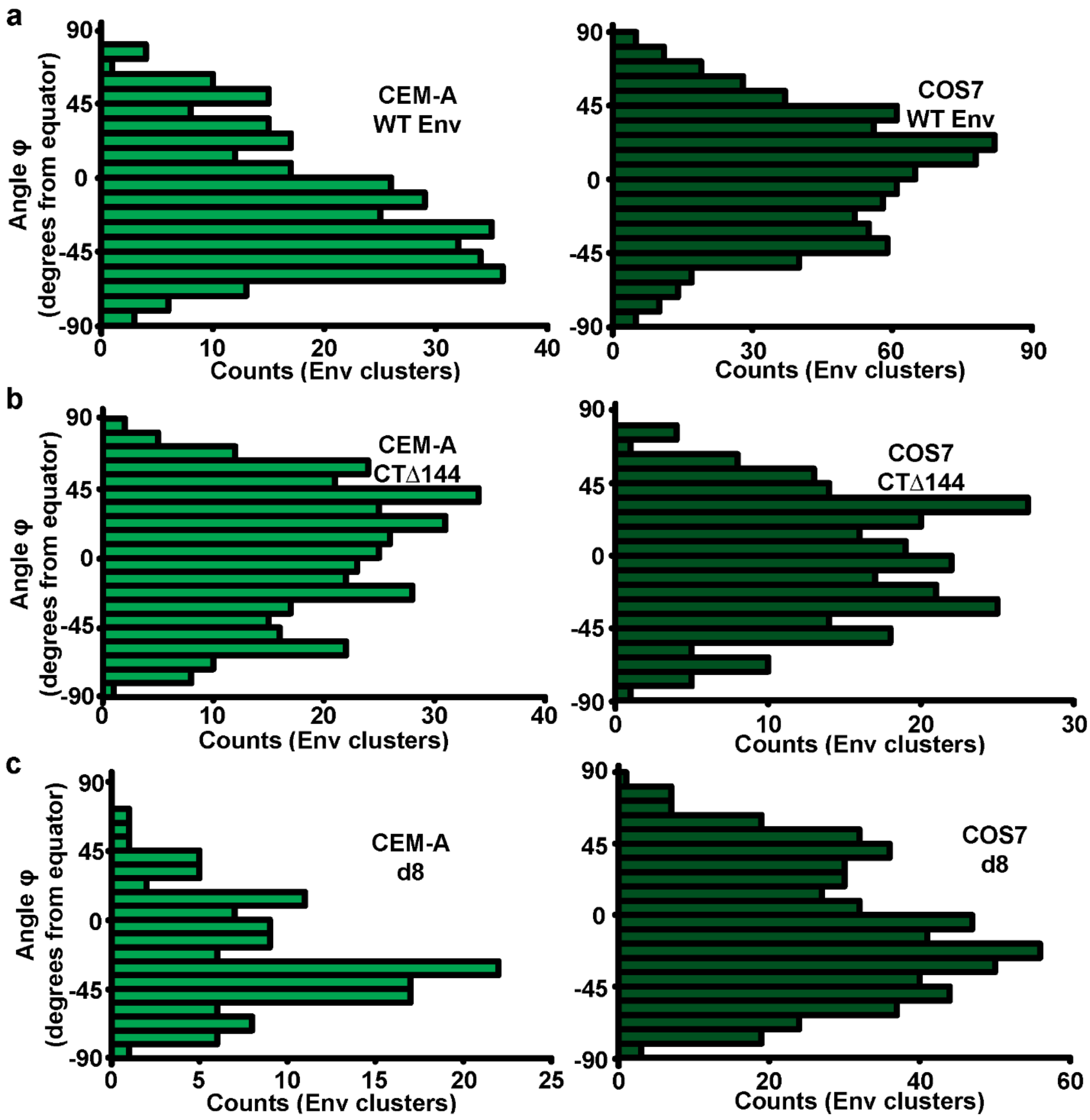

Supplementary Fig. 14. Angular distributions of Env measured by segmentation of individual clusters yields trends identical to single particle averaging of assembly sites. Individual clusters were segmented using 3D convolution and template matching. The elevation angle φ was calculated implicitly for each cluster. Distributions of φ were binned in 9° increments as in Fig. S13. **(a)** Distribution of WT-Env clusters is neck-biased in CEM-A (left, n = 338 clusters on 265 particles from 3 cells) and unbiased on COS7 cells (right, n = 813 clusters on 454 particles from 5 cells). **(b)** Distribution of CTΔ144-Env clusters is unbiased on both CEM-A (left, n = 367 clusters on 276 particles from 4 cells) and COS7 cells (right, n = 260 clusters on 244 particles from 3 cells). **(c)** Distribution of d8-Env clusters is neck-biased when produced in both CEM-A (left, n = 134 clusters on 124 particles from 4 cells) and COS7 cells (right, n = 582 clusters on 474 particles from 4 cells).

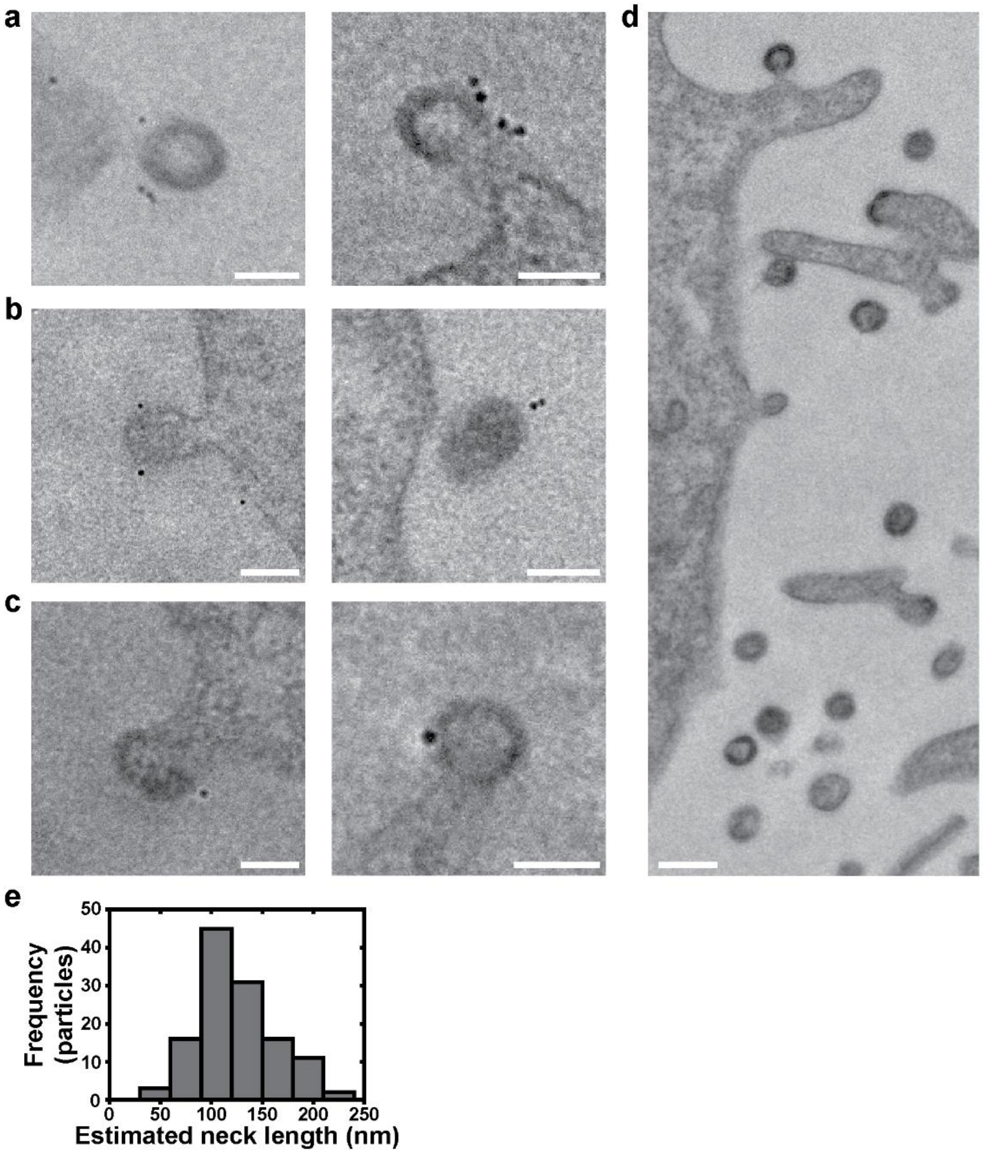

Supplementary Fig. 15. Surface immuno-gold labeling and transmission electron microscopy imaging of Env at HIV-1 assembly sites. Only images for HIV-1 assembly sites that displayed Env labeling and visible necks are shown. Gold-proximal HIV-1 buds from CEM-A cells expressing **(a)** WT-, **(b)** CTΔ144-, and **(c)** d8-Env. CEM-A cells expressing HIV-1 Gag and Env through single-round infections were gently fixed with 4% paraformaldehyde and 0.2% glutaraldehyde and cell-surface labeled using similar conditions to those in the iPALM labeling protocol. Both anti-Env b12 (full IgG) and 2G12 were used in an attempt to saturate surface-exposed Env. Importantly, we used a 1 to 5 dilution of the stock goat anti-human antibody conjugated to 6 nm gold particles which was 4-fold in excess of the manufacturer's recommended dilution. Specific staining under these conditions yielded less than 50% of Gag budding profiles labeled with gold, but of those gold-containing assembly sites the vast majority of the budding profiles are sectioned off-axis, resulting in uncertain budding polarities. Scale bars are 100 nm **(a-c)**. **(d)** Under these optimized labeling conditions, however, no gold was detected in any fields of view for cells infected with ΔEnv virus. Scale bar is 200 nm. **(e)** Estimated mean neck lengths for transverse sections of HIV-1 budding profiles visualized by transmission electron microscopy in CEM-A cells (127±38 nm, n = 124).

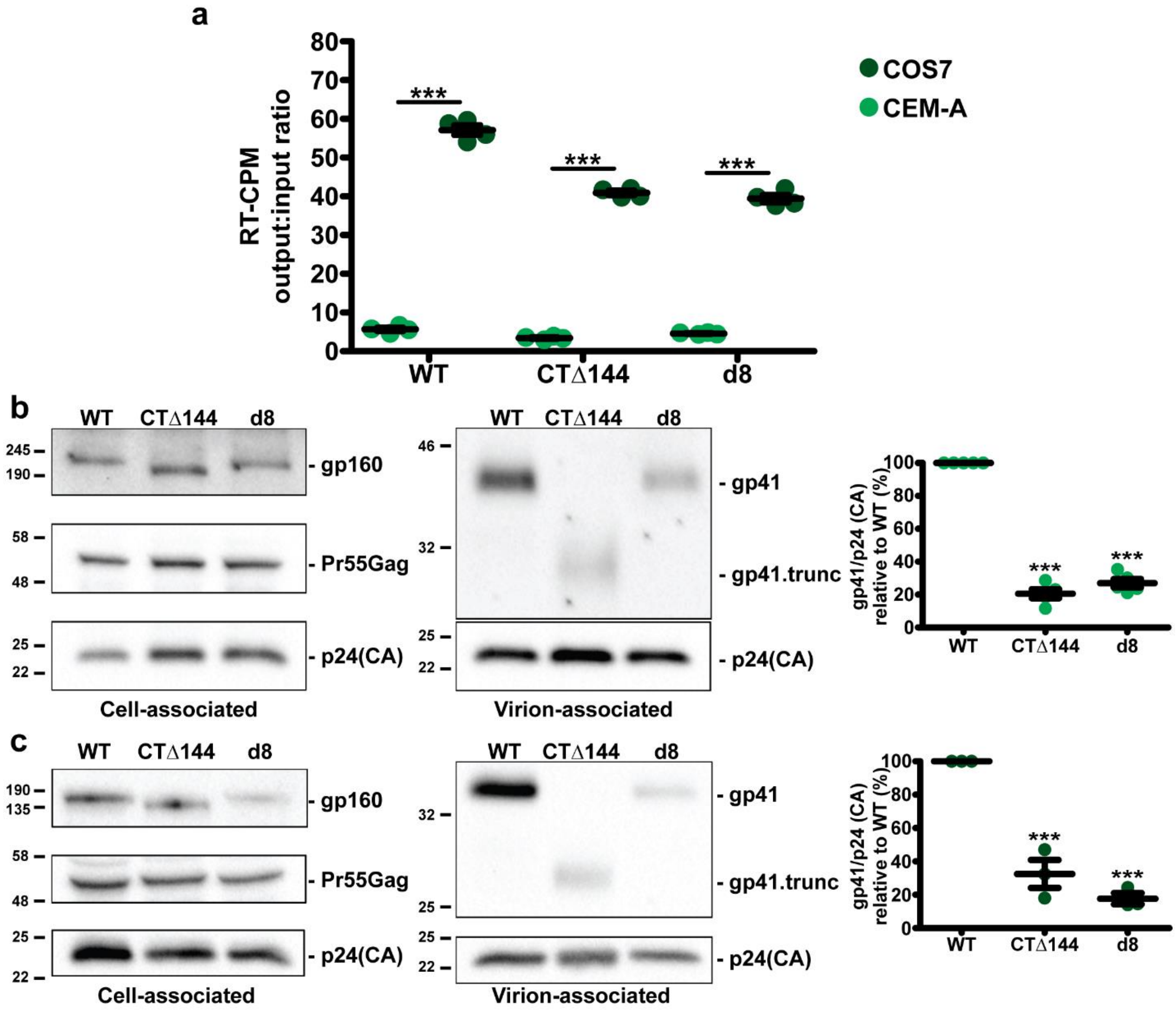

Supplementary Fig. 16. Incorporation of Env-CT mutants is defective in both CEM-A and COS7 cells, despite quantitative differences in viral protein production. (a) Ratio of output reverse transcriptase (RT) activity in the supernatant relative to input RT activity for infection, normalized to cell count, shows that infected COS7 cells produce significantly more viral protein than infected CEM-A cells. Virus produced in **(b)** CEM-A cells and **(c)** COS7 cells incorporate significantly less CTΔ144- and d8-Env relative to WT-Env. Env incorporation is calculated as the ratio of gp41 to p24 (CA) signal. Representative western blots and quantification of **(b)** 4 and **(c)** 3 independent experiments are shown. Bar and error bars represent the mean and SEM. In **(a)**, *** indicates the difference between cell lines for each Env mutant tested. In **(b)** and **(c)**, *** indicates difference from WT. For all panels, *** represents $P < 0.0001$ as determined by one-way ANOVA and Tukey's post-test.

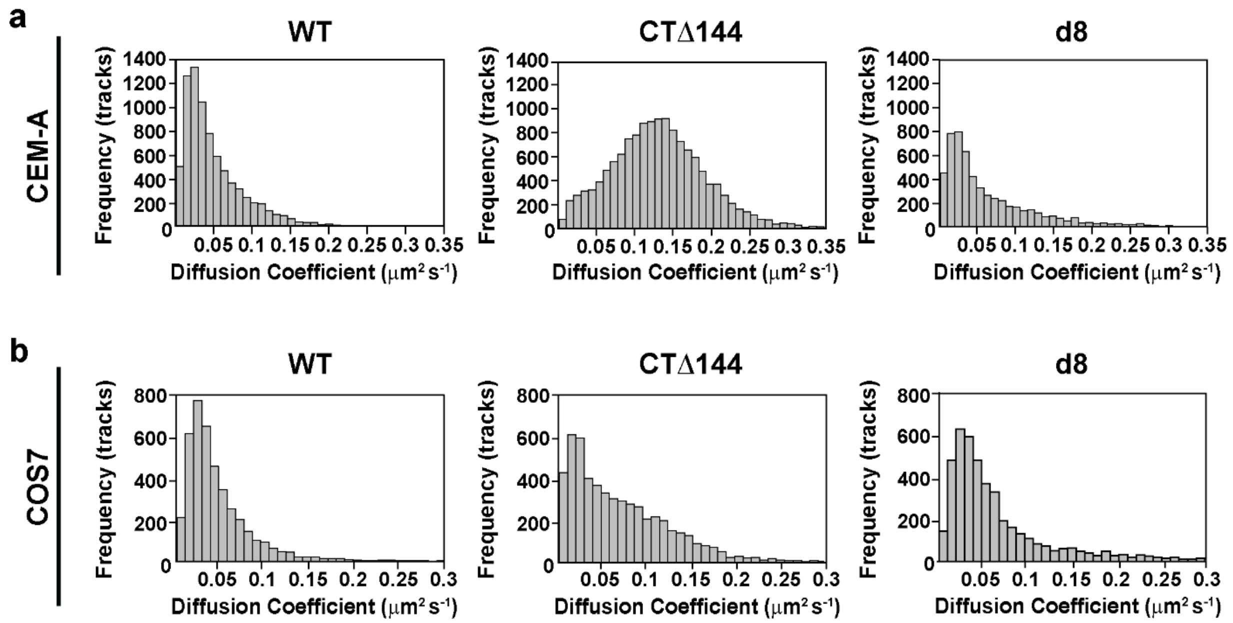

Supplementary Fig. 17. Diffusion coefficient distributions estimated from single particle tracking analysis of HIV-1 Env. The raw distributions of estimated diffusion coefficients for WT-Env, CTΔ144-Env, and d8-Env calculated using uTrack. These distributions reflect all of the obtained diffusion coefficients for each genotype and cell type irrespective of mobility classification. **(a)** CEM-A tracks (WT-Env n =7862, CTΔ144-Env n=13,338, and d8-Env n=5434) and **(b)** COS7 tracks (WT-Env n= 3790, CTΔ144-Env n=5283, and d8-Env n=4384) obtained by single particle tracking.

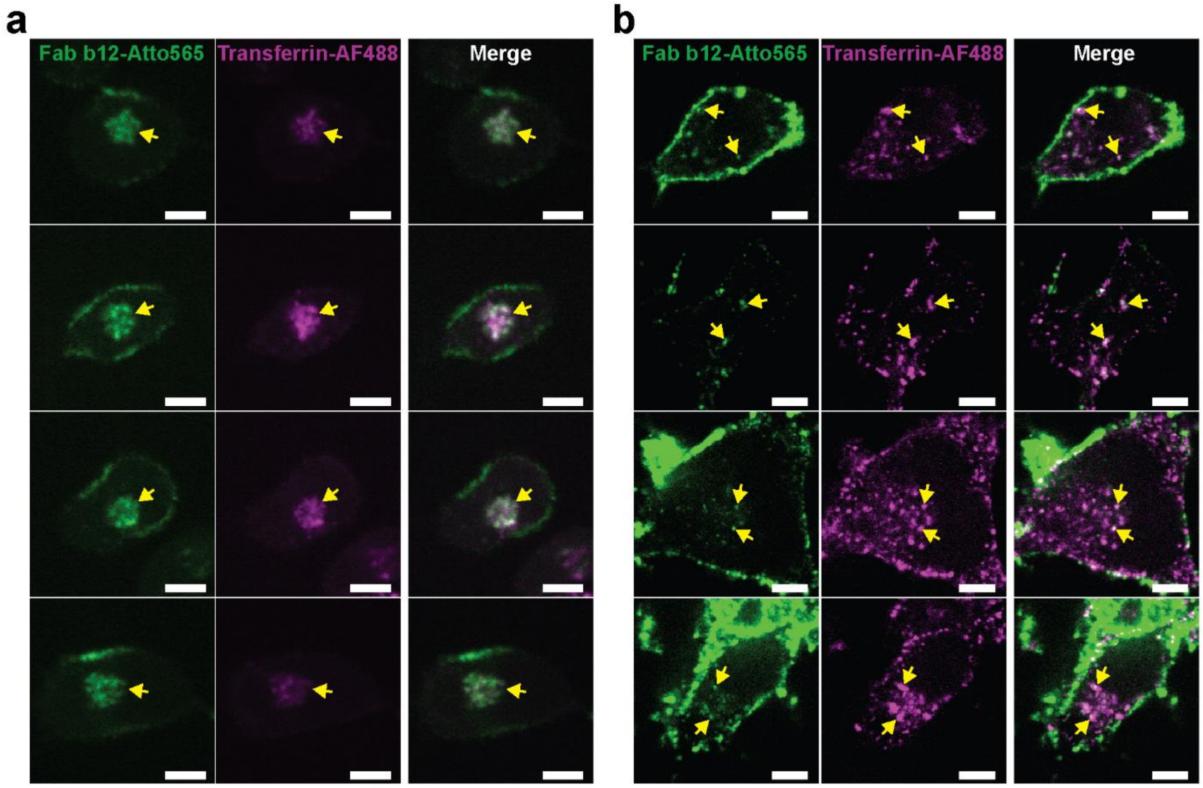

Supplementary Fig. 18. Surface labeled Env is endocytosed and colocalizes with internalized transferrin. Living cells were simultaneously pulse-chase labeled with anti-Env Fab b12-Atto565 and Transferrin-AF488 for 12 minutes. Trafficking was halted using 4% paraformaldehyde and 0.2% glutaraldehyde. **(a)** Single-round infected CEM-A cells showed colocalization of Env (left, green) with labelled transferrin (middle, magenta) that had been internalized over the chase phase of the experiment. This strong colocalization identifies the Env intracellular compartment as the recycling endosome. Scale bars are 10 µm. **(b)** COS7 cells expressing WT-Env (left, green) were labeled by the same method as above. Intracellular Env was markedly less consolidated in a centralized compartment when qualitatively compared to intracellular Env in CEM-A, but still showed colocalization (yellow arrows) with intracellular transferrin (middle, magenta). Scale bars are 20 µm.

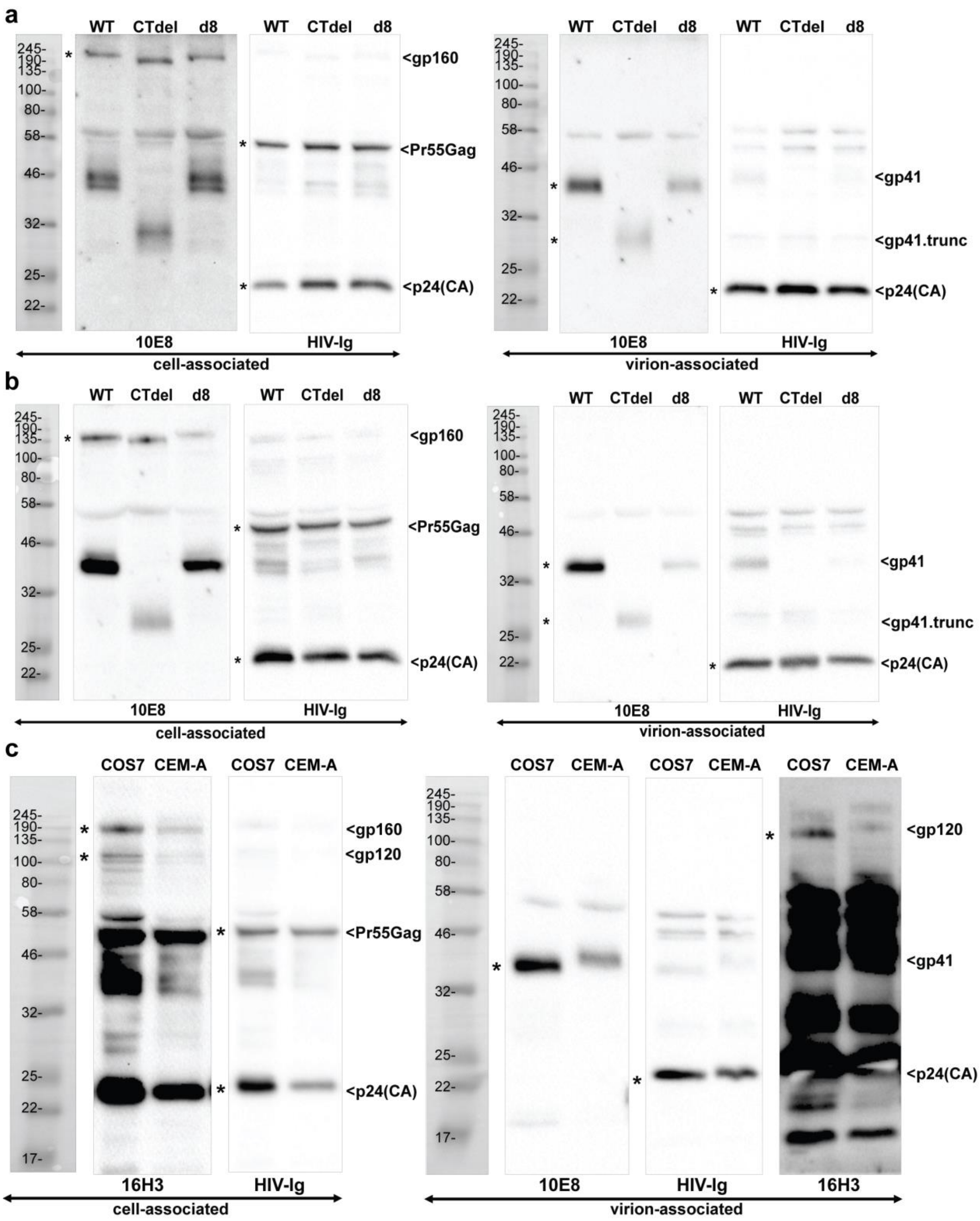

Supplementary Fig. 19. Western blots demonstrating relative levels of HIV-1 Gag and Env. (a) Western blot detection of HIV-1 Env and Gag produced in CEM-A cells. **(b)** Western blot detection of HIV-1 Env and Gag in COS7 cells. **(c)** Direct comparison of HIV-1 Env and Gag production in CEM-A versus COS7 cells. Full blots from Fig. 6 and Fig. S16. Antibodies 10E8 and 16H3 detect HIV-1 Envelope. Antibodies directed against HIV-1 Gag detect multiple domains of Gag (HIV-Ig).

IPALM EXPERIMENTS	CEM-A WT-ENV	CEM-A CTΔ144-ENV	CEM-A D8-ENV	COS7 WT-ENV	COS7 CTΔ144-ENV	COS7 D8-ENV
**ENV LOCALIZATIONS**	27,842	33,070	16,031	83,906	46,336	64,574
**ENV CLUSTERS**	338	367	134	813	260	582
**PARTICLES**	265	276	124	454	244	474
**CELLS**	3	4	4	5	3	4
**INDEPENENT EXPERIMENTS**	2	3	3	3	2	3

Supplementary Table 1. All n values of iPALM datasets for all conditions.

Supplementary References

1 Zhu, P. *et al.* Electron tomography analysis of envelope glycoprotein trimers on HIV and simian immunodeficiency virus virions. *Proceedings of the National Academy of Sciences of the United States of America* 100, 15812-15817, doi:10.1073/pnas.2634931100 (2003).

2 Lehmann, M. *et al.* Quantitative multicolor super-resolution microscopy reveals tetherin HIV-1 interaction. *PLoS pathogens* 7, e1002456, doi:10.1371/journal.ppat.1002456 (2011).

3 Chojnacki, J. *et al.* Maturation-dependent HIV-1 surface protein redistribution revealed by fluorescence nanoscopy. *Science* 338, 524-528, doi:10.1126/science.1226359 (2012).
